# Identification of favorable SNP alleles and candidate genes for traits related to early maturity via GWAS in upland cotton

**DOI:** 10.1186/s12864-016-2875-z

**Published:** 2016-08-30

**Authors:** Junji Su, Chaoyou Pang, Hengling Wei, Libei Li, Bing Liang, Caixiang Wang, Meizhen Song, Hantao Wang, Shuqi Zhao, Xiaoyun Jia, Guangzhi Mao, Long Huang, Dandan Geng, Chengshe Wang, Shuli Fan, Shuxun Yu

**Affiliations:** 1 College of Agronomy, Northwest A&F University, Yangling, China; 2 State Key Laboratory of Cotton Biology, Institute of Cotton Research of the Chinese Academy of Agricultural Sciences, Anyang, China; 3 Bioinformatics Division, Biomarker Technologies Corporation, Beijing, China; 4 Cotton Research Institute, Xinjiang Academy of Agricultural and Reclamation Science, Shihezi, Xinjiang China

**Keywords:** *Gossypium hirsutum* L, GWAS, SLAF-seq, Early maturity traits, Candidate gene, SNP alleles

## Abstract

**Background:**

Early maturity is one of the most important and complex agronomic traits in upland cotton (*Gossypium hirsutum* L). To dissect the genetic architecture of this agronomically important trait, a population consisting of 355 upland cotton germplasm accessions was genotyped using the specific-locus amplified fragment sequencing (SLAF-seq) approach, of which a subset of 185 lines representative of the diversity among the accessions was phenotypically characterized for six early maturity traits in four environments. A genome-wide association study (GWAS) was conducted using the generalized linear model (GLM) and mixed linear model (MLM).

**Results:**

A total of 81,675 SNPs in 355 upland cotton accessions were discovered using SLAF-seq and were subsequently used in GWAS. Thirteen significant associations between eight SNP loci and five early maturity traits were successfully identified using the GLM and MLM; two of the 13 associations were common between the models. By computing phenotypic effect values for the associations detected at each locus, 11 highly favorable SNP alleles were identified for five early maturity traits. Moreover, dosage pyramiding effects of the highly favorable SNP alleles and significant linear correlations between the numbers of highly favorable alleles and the phenotypic values of the target traits were identified. Most importantly, a major locus (*rs13562854*) on chromosome D_t_3 and a potential candidate gene (*CotAD_01947*) for early maturity were detected.

**Conclusions:**

This study identified highly favorable SNP alleles and candidate genes associated with early maturity traits in upland cotton. The results demonstrate that GWAS is a powerful tool for dissecting complex traits and identifying candidate genes. The highly favorable SNP alleles and candidate genes for early maturity traits identified in this study should be show high potential for improvement of early maturity in future cotton breeding programs.

**Electronic supplementary material:**

The online version of this article (doi:10.1186/s12864-016-2875-z) contains supplementary material, which is available to authorized users.

## Background

Cotton is the most important natural textile fiber source worldwide. The tetraploid species *Gossypium hirsutum* L. (2n = 4x = 52, AD genome), also referred to as ‘upland cotton’, accounts for 95 % of the world’s cotton production. Early fiber production is one of the most important traits in cotton, and the selection and popularization of early-maturing cotton varieties are of significant value in reducing the dilemma of whether to plant farmlands with cotton or cereals during cropping system optimization in China [1, 2]. Early maturity is a complex quantitative trait that mainly includes components such as the growth period, growth stages (including the seedling period, squaring period, flowering and boll-setting period (FBP) and boll-opening period), yield percentage before frost (YPBF), node of the first fruiting branch (NFFB), and height of the node of the first fruiting branch (HNFFB) [1, 2]. These components of this quantitative trait are regulated by quantitative trait loci (QTLs) and the environment, as reflected in different genetic models in different cultivars [3]. Early maturity has been reported to be negatively correlated with yield and fiber quality [3]. It is difficult to simultaneously improve early maturity, yield and fiber quality using conventional breeding methods. Fortunately, the rapid development of applied genomics research has provided alternative tools to improve efficiency in plant breeding programs. For example, molecular markers linked to causal genes or QTLs can be used for marker-assisted selection (MAS) and genomic selection.

Over the last two decades, many QTLs related to target traits have been identified using QTL-mapping methods by constructing intraspecific segregating populations of *G. hirsutum* with different target traits, such as fiber quality traits [4–6], yield and its components [7], resistance traits [8–10], early maturation traits [2, 11, 12] and drought-related traits [13]. In a study of traits associated with early maturity in cotton, more than 70 related QTLs were detected by linkage mapping [2, 11, 12]. These QTLs may be valuable for improving early maturity by MAS.

Association mapping is another effective approach for connecting phenotypes and genotypes in plants when information on population structure and linkage disequilibrium (LD) is available [14]. This method is convenient because it helps to avoid the difficulty of screening large biparental mapping populations. Association mapping was introduced to maize genetics in 2001 [14] and has been subsequently applied in studies of many plant species [15]. Association mapping is widely used to identify molecular markers associated with target traits, and it has been employed in genetic studies of rice, maize, wheat and other important agricultural crops [16–19]. Genome-wide association studies (GWAS) represent a powerful approach for identifying the locations of genetic factors that underlie complex traits [20]. GWAS have been successfully implemented in *Arabidopsis thaliana* [21, 22], rice [20, 23], maize [24] and soybean [25] for the identification of single nucleotide polymorphism (SNP) loci and candidate genes for various ecological and agricultural traits. In recent years, association mapping has also been widely used in studies of cotton [10, 19, 26–30]. For example, Abdurakhmonov et al. [19] performed association mapping to examine QTLs related to fiber-quality traits in *G. hirsutum* accessions using microsatellite markers. Further, Kantartzi and Stewart [26] detected QTLs related to fiber quality in *G. arboreum* accessions using association mapping with simple sequence repeat (SSR) markers. Recently, Association mapping was performed to assess QTL alleles during three cotton breeding periods, revealing that some alleles could be detected in nearly all of the Chinese cotton cultivars studied [29]. Favorable QTL alleles for yield and its components have been identified via association mapping in Chinese upland cotton cultivars [28]. Some QTL alleles associated with verticillium wilt resistance in upland cotton have also been detected using this approach [10]. However, few QTLs for cotton early maturity traits have been identified via association mapping.

To better understand the genetic architecture of early maturity traits in upland cotton, genome-wide SNP discovery based on the specific-locus amplified fragment sequencing (SLAF-seq) method and a GWAS strategy were used to identify the SNP loci associated with early maturity traits. We successfully identified several significant associations between SNP loci and early maturity traits using the generalized linear model (GLM) and mixed linear model (MLM). The highly favorable SNP alleles for early maturity traits were mined by computing the phenotypic effect of each SNP locus identified, and the pyramiding effects of the highly favorable SNP alleles for these traits were assessed. Moreover, major SNP loci and potential candidate genes for early maturity were detected. The results of this important study serve as a foundation for analyses of the genetic mechanisms underlying cotton earliness and for MAS for early maturity in cotton.

## Results

### Genome and chromosome characteristics of SLAF-based SNPs in upland cotton varieties

**Fig. 1:**
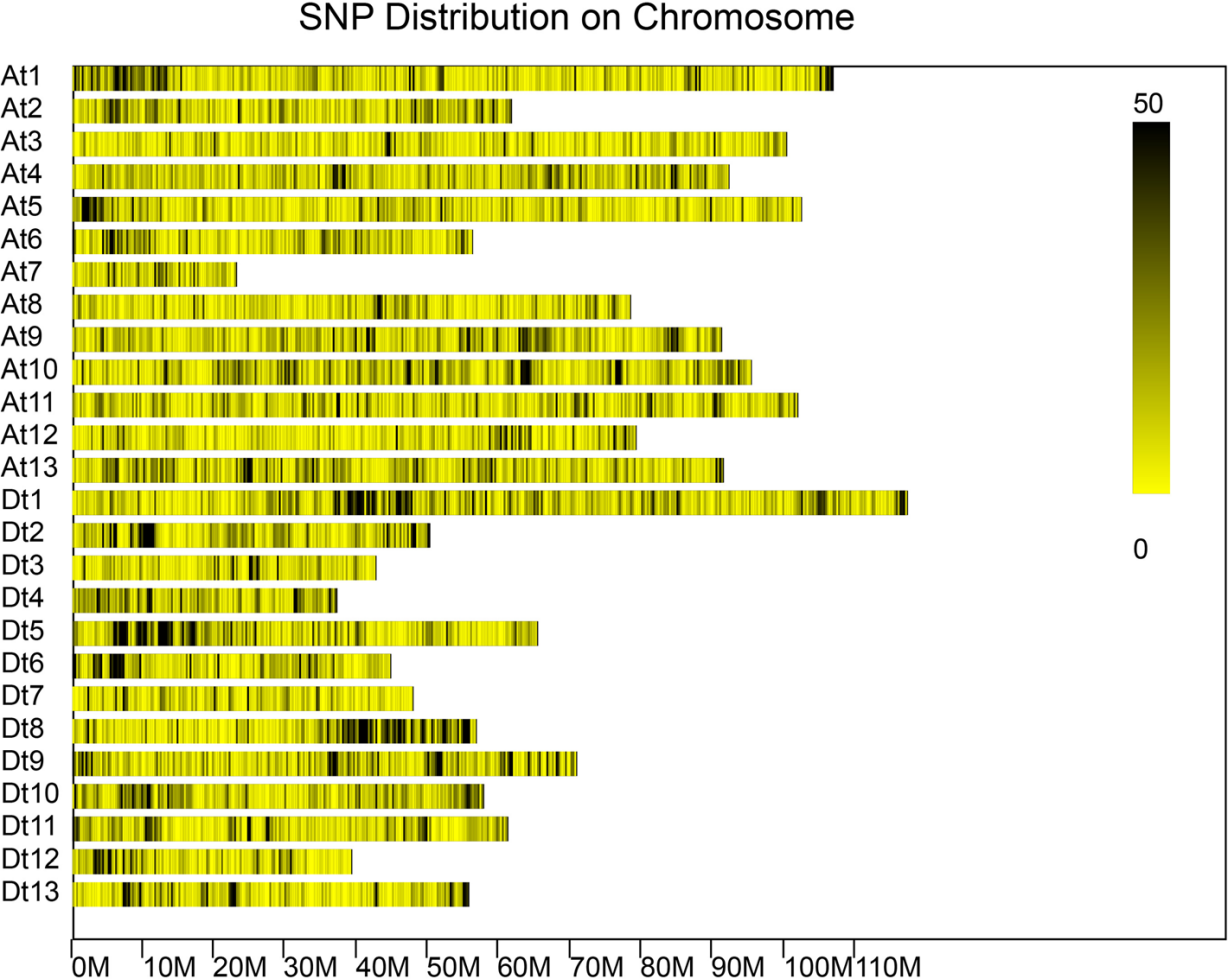
Single nucleotide polymorphism (SNP) distributions on the 26 chromosomes of upland cotton. A_t_1 ~ A_t_13 and D_t_1 ~ D_t_13 in vertical axis are the serial number of 26 chromosomes; The horizontal axis shows chromosome length (Mb); 0 ~ 50 depicts SNP density (the number of SNPs per window)

SLAF-seq was performed with an Illumina HiSeq 2500 (Illumina, Inc.; San Diego, CA, US) at Biomarker Technologies Corporation in Beijing to genotype 355 cotton varieties/accessions. The sequencing run generated 96.10 Gb of data, including 874.44 million paired-end reads with an length of ~80 bp. The Q30 ratio and guanine-cytosine (GC) content, which are indicators of sequencing quality, were 89.75 and 39.11 %, respectively, indicative of good quality. A total of 678,397 high-quality SLAF tags were obtained for each of the 355 genotypes, and 505,823 polymorphic SLAFs were identified from these reads by performing sequence alignments with the TM-1 reference genome [31]. The SLAFs, which had an average depth of 5.39-fold per sample among the 355 varieties/accessions, were used for calling SNPs. A total of 691,978 SNPs were initially called for the 355 genotypes (Fig. 1). SNP loci with a minor allele frequency (MAF) of <5 % cannot be used in association analyses; thus, most of the SNPs (88.20 %) were removed, and the remaining 81,675 SNPs with an MAF ≥0.05 were used in subsequent analyses.

The 81,675 SNP markers covered all 26 chromosomes. The largest number of markers was identified on chromosome D_t_1 (5882 SNPs), and the smallest was identified on chromosome A_t_7 (1006 SNPs). The average marker density was approximately one SNP per 24.85 kb. The highest marker density was detected on chromosome D_t_8 (one SNP per 15.76 kb), and the smallest was identified on chromosome A_t_3 (one SNP per 36.24 kb) (Fig. 1, Table 1).

**Table 1:** SNP distribution on each chromosome

Chromosome	SNP number	Chromosome length (Mb)	SNP density^a^ (kb)	Chromosome	SNP number	Chromosome length (Mb)	SNP density^a^ (kb)
A_t_1	4553	106.99	23.50	D_t_1	5882	117.24	19.93
A_t_2	2890	61.75	21.37	D_t_2	2801	50.19	17.92
A_t_3	2769	100.35	36.24	D_t_3	1464	42.80	29.23
A_t_4	4207	92.29	21.94	D_t_4	2166	37.36	17.25
A_t_5	3688	102.56	27.81	D_t_5	3903	65.57	16.80
A_t_6	2721	56.48	20.76	D_t_6	2378	44.80	18.84
A_t_7	1006	23.27	23.13	D_t_7	1480	47.98	32.42
A_t_8	2540	78.75	31.00	D_t_8	3611	56.89	15.76
A_t_9	4268	91.12	21.35	D_t_9	3774	70.91	18.79
A_t_10	4877	95.48	19.58	D_t_10	3026	57.80	19.10
A_t_11	4032	102.02	25.30	D_t_11	2567	61.12	23.81
A_t_12	2564	79.16	30.87	D_t_12	1658	39.38	23.75
A_t_13	4456	91.55	20.55	D_t_13	2394	55.87	23.34

^a^SNP density is presented as the average physical distance between two adjacent SNP loci

### Population structure and linkage disequilibrium

**Fig. 2:**
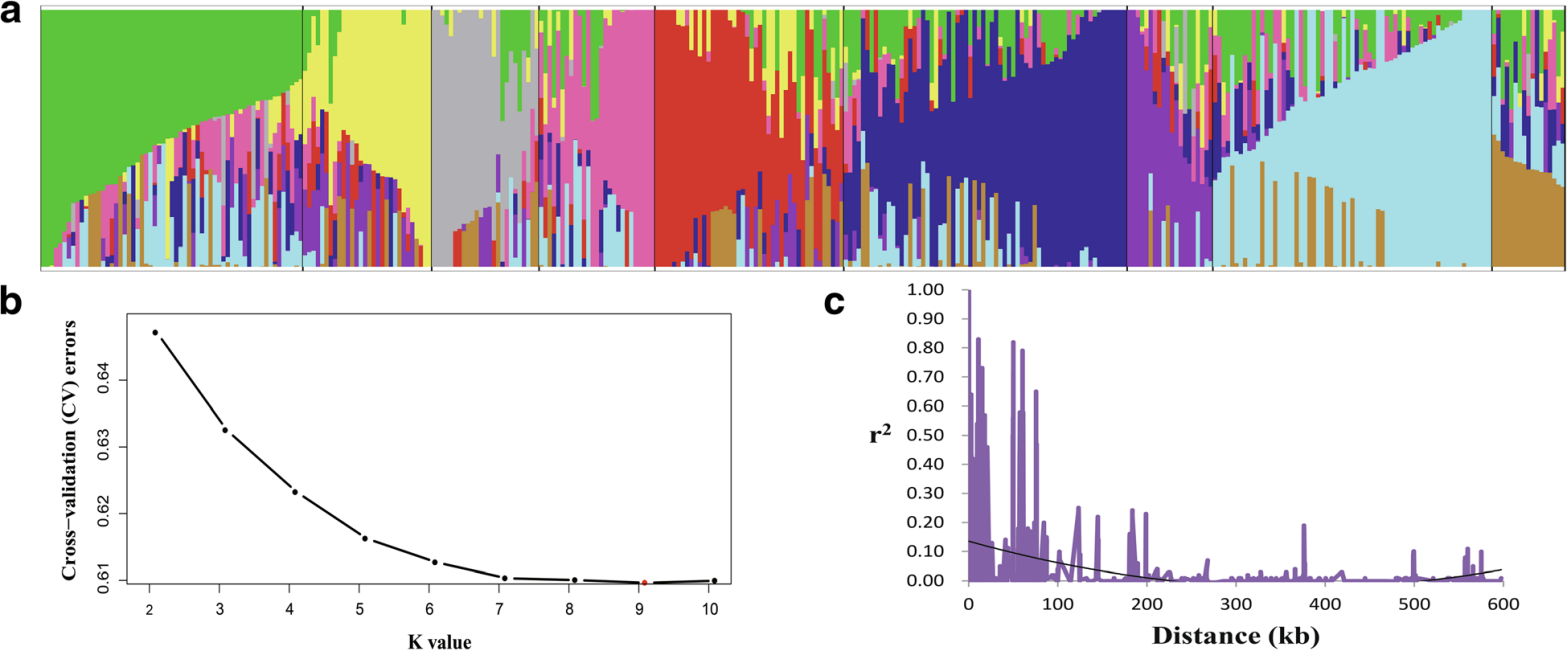
Population structure and linkage disequilibrium (LD) decay of upland cotton accessions. **a** Population structure of upland cotton accessions; *each line* is represented by a single *vertical line*, and *each color* represents one cluster; **b** estimated ln(cross-validation errors in the data) calculated for K, ranging from 2 to 10; **c** the mean LD decay rate was estimated as the squared correlation coefficient (r^2^) using all pairs of SNPs located within 600 kb of physical distance in genomic regions in a population of 355 upland cotton germplasm accessions

To estimate the number of subgroups in the population of 355 upland cotton accessions, structure analysis was performed using 81,675 SNPs from the 355 accessions. The results indicated that the minimum number of cross-validation errors was K = 9, which was thus determined to be the optimum K, and that the testing accessions could be separated into nine subpopulations (Fig. 2a, b). Subpopulations 1–9 included 60, 30, 25, 27, 45, 66, 20, 65, and 17 accessions, respectively. To represent the genetic diversity among the 355 accessions, a total of 185 upland cotton lines were screened, which included approximately 50 % of the accessions of each of the subpopulations, taking into consideration the diverse geographic origins and maturity traits. A total of 32, 16, 13, 15, 24, 35, 12, 30 and 8 lines were selected from each of the subpopulations 1–9, respectively. Most of these upland cotton accessions from each subpopulation had mixed ancestry, and the obvious geographic subpopulation was not found, indicating that these lines might have experienced introgression or gene flow during cotton breeding in China.

To determine the mapping resolution for GWAS, we quantified the average extent of LD decay. Using the whole set of SNPs, the LD decay rate of the population for the entire genome was estimated to be 100 kb, with *r*^2^ = 0.07 at half of the maximum value (Fig. 2c).

### Phenotypic characteristics of traits related to early maturity

**Fig. 3:**
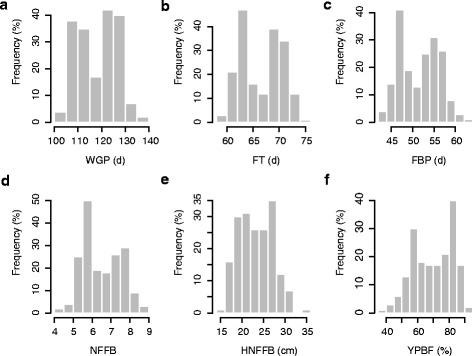
Frequency distributions of the mean values of six maturity traits of 185 cotton accessions in four environments. **a** whole growth period (WGP); **b** flowering time (FT); **c** flowering and boll-setting period (FBP); **d** node of the first fruiting branch (NFFB); **e** height of the node of the first fruiting branch (HNFFB); and **f** yield percentage before frost (YPBF)

A core set of 185 upland cotton lines was selected for association analysis based on analysis of the population structure, and the traits of these lines related to early maturity were investigated across four field environments. The mean whole growth period (WGP) durations were 116.61, 117.92, 118.03 and 120.39 d in the four experiments, respectively. The minimum WGP was 96.67 d in SU-2013, and the maximum WGP was 147.00 d in SP-2014. Analogously, the FT and FBP exhibited wide ranges of 53.00–80.67 d and 38.00–73.67 d, with means of 66.59 and 51.64 d, respectively. The NFFB ranged from 3.00 to 12.00, with a mean of 6.50. The mean HNFFB values exhibited continuous variation, ranging from 15.45 to 34.03 cm. The YPBF exhibited the largest range of variation, ranging from 1.55 to 100 %. The mean coefficients of variance (CVs) for the WGP, FT, FBP, NFFB, HNFFB and YPBF were 6.88, 5.91, 8.79, 15.79 16.92 and 18.11 %, respectively. These data indicated a high degree of diversity in early maturity phenotypic traits in the natural population. Based on the WGP, the number of early-maturing accessions (106 d < WGP ≤112 d), early-middle-maturing accessions (114 d < WGP ≤120 d) and middle-late-maturing accessions (122 d < WGP ≤128 d) were 62 (33.51 %), 20 (10.81 %) and 59 (31.89 %), respectively. The early-middle-maturing accessions accounted for a very small percentage, thus these traits were typically bimodally distributed (Fig. 3, Additional file 1: Table S1).

Analysis of variance (ANOVA) indicated that the genotype (G) and interactions between the genotype and environmental factors (G × E) were both significant (*P* < 0.01) for all six traits (Additional file 1: Table S1). The correlation coefficients for the association of the WGP with the FT, FBP, NFFB, HNFFB and YPBF were 0.9541, 0.9659, 0.8775, 0.8513 and −0.9230, respectively. These results indicated that the WGP was significantly associated with the FT, FBP, NFFB, HNFFB and YPBF in all four environments (*P* < 0.01) (Additional file 1: Table S2).

### GWAS for early maturity traits

**Fig. 4:**
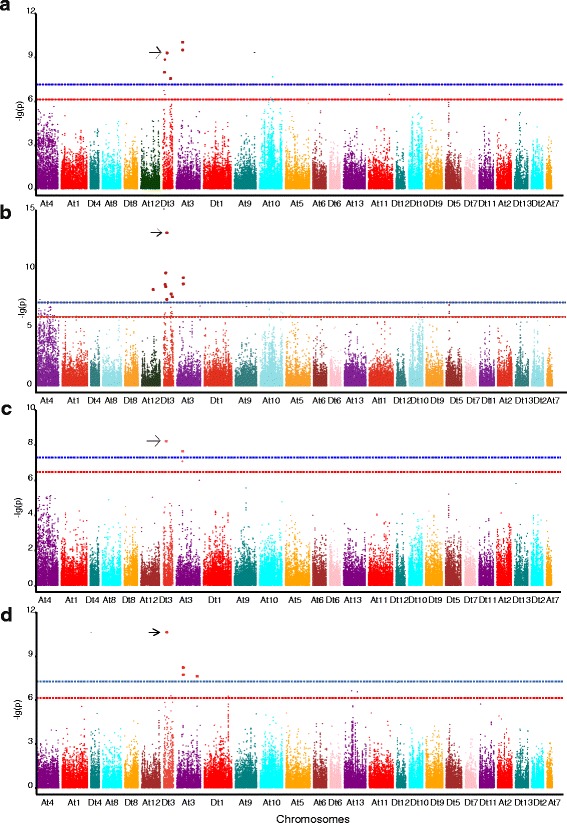
Manhattan plots of genome-wide association studies (GWAS) for the WGP (**a**), FT (**b**), NFFB (**c**) and YPBF (**d**) measured with the generalized linear model (GLM) using the best linear unbiased prediction (BLUP) values for the four environments. The SNP locus *rs13562854* is indicated by the *black arrow*. The general and highly significant trait-associated SNPs are distinguished by the *red* and *blue* threshold lines, respectively

**Fig. 5:**
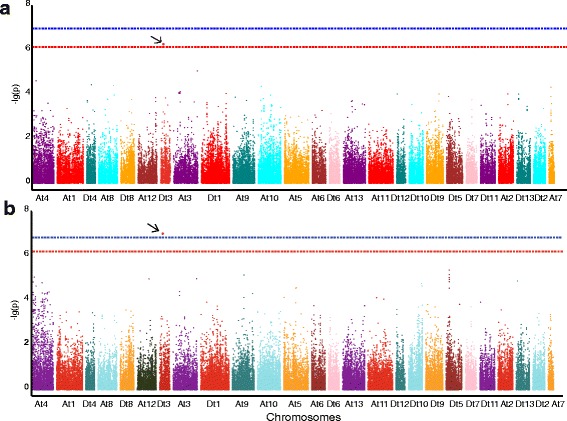
Manhattan plots of genome-wide association studies (GWAS) for the WGP (**a**) and FT (**b**) measured with the mixed linear model (MLM) using the best linear unbiased prediction (BLUP) values for the four environments. The SNP locus *rs13562854* is indicated by the *black arrow*. The general and highly significant trait-associated SNPs are distinguished by the *red* and *blue* threshold lines, respectively

To investigate the genotypic variation that underlies the traits related to early maturity in cotton, GWAS was performed to identify the associated SNP loci in upland cotton accessions. In the GLM, 13 associations were found to be significant between 8 SNP loci and five traits related to early maturity (all traits except for the HNFFB) according to the best linear unbiased predictions (BLUPs) and in at least two of the four environments (-lg(p) ≥6.21). Of these SNP loci, 50 % were distributed on chromosome D_t_3, and 25 % were distributed on chromosome A_t_3. Among these associations, five associations each with the WGP and FT were identified, as well as one association each with the FBP, NFFB and YPBF; the corresponding SNP loci were distributed on chromosome D_t_3. The SNP loci for various early maturity traits identified through GWAS explained 5.36–15.56 % of the phenotypic variance (Additional file 1: Table S3, Fig. 4 and Additional file 2: Figure S1 and Additional file 3: Figure S2). Among these associated SNP loci, three were co-associated with two or more different traits. For example, *rs13562854* (D_t_3) was simultaneously associated with the WGP, FT, NFFB and YPBF (Additional file 1: Table S3, Fig. 4). The MLM results indicated that two associations were significant between one SNP locus and two traits (-lg(p) ≥ 6.21), i.e., one SNP locus (*rs13562854*) on chromosome D_t_3 was found to be simultaneously associated with the WGP and FT according to BLUPs and in two of the four environments, explaining 9.23–16.46 % of the phenotypic variance (Additional file 1: Table S3, Fig. 5 and Additional file 4: Figure S3 and Additional file 5: Figure S4). It was very important and meaningful that the SNP locus *rs13562854* was simultaneously associated with the WGP and FT and was detected via both the GLM and MLM (Additional file 1: Table S3, Figs. 4 and 5).

### Mining of highly favorable SNP alleles associated with early maturity traits

In our study, SNP alleles with positive effects that led to decreases in the WGP, FT, FBP, NFFB and HNFFB or an increase in the YPBF were defined as “favorable alleles”, and those that resulted in increases in the WGP, FT, FBP, NFFB and HNFFB or a decrease in the YPBF were defined as “unfavorable alleles”. Among the favorable SNP alleles, *rs26538646* (tightly linked with *rs26538688*), *rs13562854*, *rs8917898* and *rs13153008* had the strongest positive phenotypic effects on the WGP, shortening it by 6.70 d, 7.53 d, 7.58 d and 7.76 d, respectively; in addition, *rs22465987*, *rs48627288*, *rs13562854*, *rs8917898* and *rs37255056* shortened the FT by 0.07 d, 0.55 d, 3.88 d, 3.69 d and 3.40 d, respectively; *rs13153008* shortened the FBP by 4.09 d; and *rs13562854* shortened the NFFB by 0.91, whereas it increased the YPBF by 10.45 %. These findings indicated that the phenotypic characteristics of the genotypes with favorable SNP alleles were significantly enhanced compared with those of the genotypes with unfavorable SNP alleles, with the exception of *rs22465987* and *rs48627288* (ANOVA; *P* < 0.01). The highly favorable SNP alleles exhibited significantly different traits compared with the unfavorable alleles (*P* < 0.01). Finally, the eleven highly favorable SNP alleles were mined by ANOVA. The numbers of highly favorable SNP alleles for the WGP, FT, FBP, NFFB and YPBF were 5, 3, 1, 1 and 1, respectively (Table 2).

**Table 2:** Favorable SNP alleles, their phenotypic effects (a_i_), and representative accessions

Traits	SNP	Position	Alleles	Favorable alleles	a_i_	Accessions	Representative accessions^a^
WGP	*rs26538646*	A_t_3:26538646	A/G	A	−6.70^**^	59	zhongmiansuo74, xia25, zhong416
	*rs26538688*	A_t_3:26538688	G/T	G	−6.70^**^	59	zhongmiansuo74, xia25, zhong416
	*rs13562854*	D_t_3:13562854	A/G	A	−7.53^**^	66	zhongmiansuo74, xia25, zhong416
	*rs8917898*	D_t_3:8917898	A/G	G	−7.58^**^	49	zhong6426, zhong51822, xia13-7
	*rs13153008*	D_t_3:13153008	A/G	A	−7.76^**^	42	xia25, zhong416, baimian17
FT	*rs22465987*	A_t_4:22465987	A/G	G	−0.07	94	1476, zhongmiansuo74, xiaomian3
	*rs48627288*	A_t_12:48627288	A/G	A	−0.55	86	xia25, xiazao3, zhongmiansuo14
	*rs13562854*	D_t_3:13562854	A/G	A	−3.88^**^	66	xiazao2,zhongmiansuo74,xia25
	*rs8917898*	D_t_3:8917898	A/G	G	−3.69^**^	49	xia25, 1476, xiazao3
	*rs37255056*	D_t_3:37255056	A/G	A	−3.40^**^	56	xia25, 1476, xiazao3
FBP	*rs13153008*	D_t_3:13153008	A/G	A	−4.09^**^	42	zhong416, xia25, zhongmiansuo64
NFFB	*rs13562854*	D_t_3:13562854	A/G	A	−0.91^**^	66	xiaozao2, xiazao3, xia25
YPBF	*rs13562854*	D_t_3:13562854	A/G	A	10.45^**^	66	zhongmiansuo74, xia25, xiazao3

^a^Representative accessions consist of the top 3 entries for the target trait values of accessions with the corresponding favorable alleles; ^**^highly favorable SNP alleles that exhibit significantly different traits compared with the unfavorable alleles (*P* < 0.01)

### Pyramiding effects of highly favorable SNP alleles associated with early maturity traits

To determine whether the highly favorable SNP alleles for traits related to early maturity had pyramiding effects, the mean WGP, FT, FBP, NFFB and YPBF values of the accessions that contained different numbers of highly favorable SNP alleles were analyzed by ANOVA. The results indicated that earlier maturation occurred in the cotton accessions with the highly favorable SNP alleles compared with those without these alleles, as well as those with fewer of these alleles (Table 3). For example, the average WGP of the genotypes without highly favorable alleles was 125.05 d, that of those with a single highly favorable allele was 117.39 d, that of those with two highly favorable alleles was 113.55 d, and that of those with four highly favorable alleles was 108.84 d.

**Table 3:** Pyramiding effects of the highly favorable alleles that contribute to early maturity

Traits	No. of favorable alleles	Mean ± SD	Frequency (%)
WGP (d)	0	125.05 ± 2.66 (A)	41.46
	1	117.39 ± 5.83 (B)	7.32
	2	113.55 ± 5.89 (B)	12.20
	3		
	4	108.84 ± 2.63 (C)	39.02
FT (d)	0	69.75 ± 2.26 (A)	57.48
	1	64.26 ± 1.99 (B)	11.81
	2	64.42 ± 2.36 (B)	1.57
	3	62.3 ± 1.10 (C)	29.13
FBP (d)	0	54.71 ± 3.36 (A)	39.62
	1	47.55 ± 2.1 (B)	60.38
NFFB	0	7.23 ± 0.82 (A)	59.51
	1	5.59 ± 0.38 (B)	40.49
YPBF (%)	0	61.03 ± 9.59 (A)	59.51
	1	82.19 ± 4.48 (B)	40.49

Values with different letters are significantly different (*P* < 0.05)

**Fig. 6:**
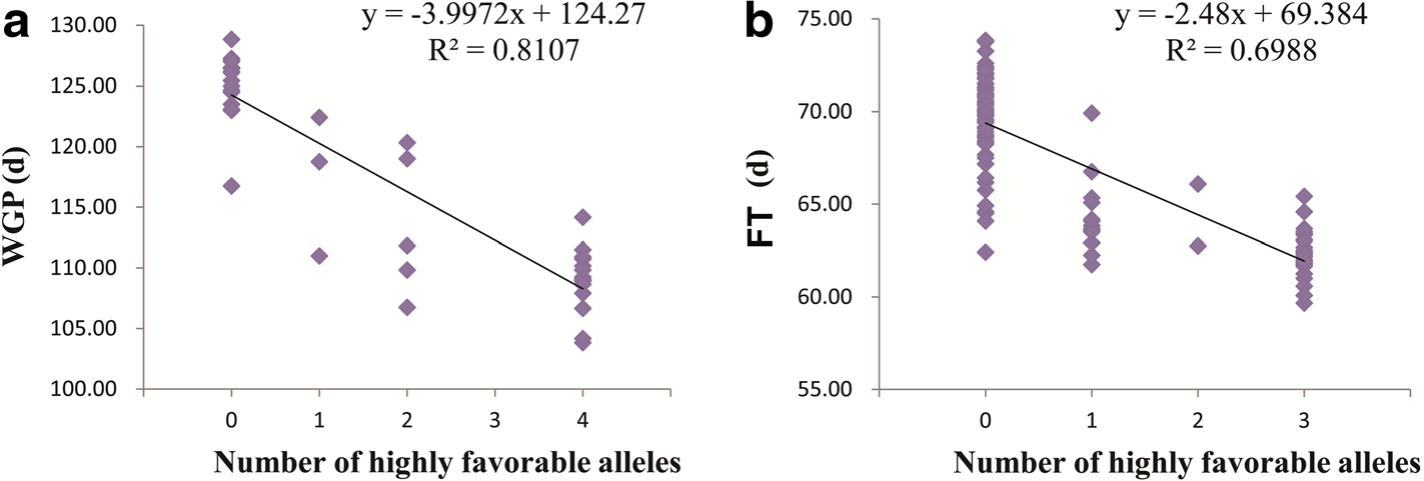
Linear regression analyses of the numbers of highly favorable SNP alleles and average WGP (**a**) and FT values (**b**) in four environments

In addition, to further assess the pyramiding effects of the highly favorable SNP alleles on the early maturity response, linear regression was conducted with the number of highly favorable SNP alleles and the average WGP and FT values for the four environments. Two significant linear correlations were detected between the WGP and number of highly favorable SNP alleles (*R*^2^ = 0.8107) and between the FT and number of highly favorable SNP alleles (*R*^2^ = 0.6988), further confirming the pyramiding effects of the highly favorable alleles (Fig. 6). These findings demonstrate that the highly favorable SNP alleles had significant pyramiding effects on the WGP and FT.

### A major locus on chromosome D_t_3 and candidate genes that potentially underlie early maturity

**Fig. 7:**
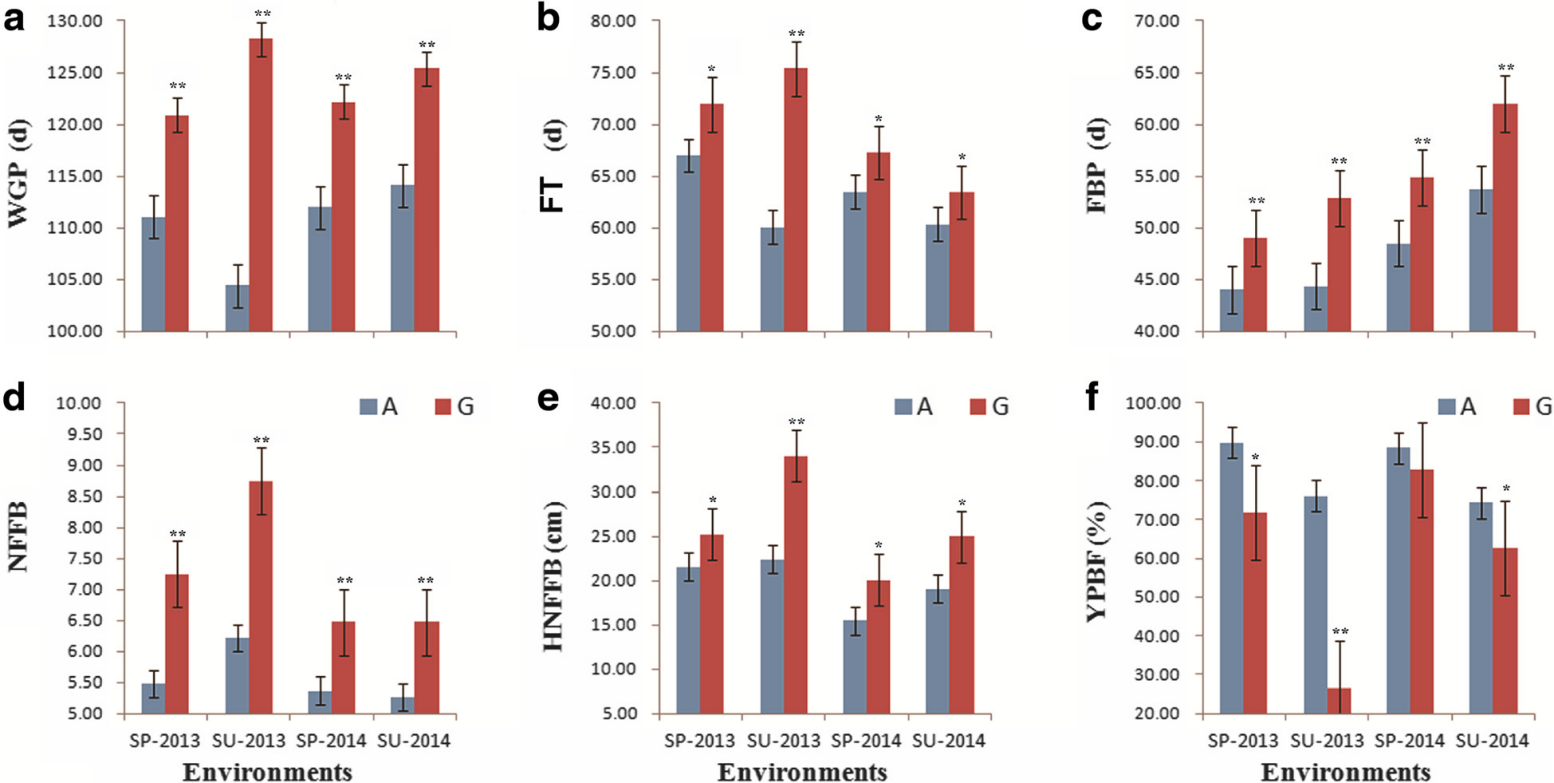
Favorable alleles (A) and unfavorable alleles (G) at the SNP locus rs13562854 for six traits related to early maturity in the four environments. **a**–**f** represent six traits related to early maturity WGP, FT, FBP, NFFB, HNFFB and YPBF, respectively; *, ** indicate significance at probability levels of 0.05 and 0.01, respectively; SP-2013, SU-2013, SP-2014 and SU-2014 are the four environments

The most favorable SNP locus (*rs13562854*) associated with both the WGP and FT in the GLM and MLM was used to compare the differences between the accessions that carried favorable alleles and those that carried unfavorable alleles in six traits related to early maturity. The mean phenotypic value of 66 accessions that contained a favorable allele (A) was significantly better (lower for the WGP, FT, FBP, NFFB and HNFFB and higher for the YPBF) compared with the remaining accessions that contained unfavorable alleles (G) (Fig. 7). This finding demonstrates that *rs13562854* on chromosome D_t_3 is a major locus for early maturity in upland cotton.

A total of 32 genes were annotated in the 1 Mb regions within 500 kb on either side of the most favorable SNP allele (*rs13562854*) (Table 4). Among these genes, definite biological function annotations could not be determined for six, and ten were annotated as putative or hypothetical proteins; among the remaining genes, 16 possessed domains of known function, and four of these 16 genes (*CotAD_01914*, *CotAD_01926*, *CotAD_01936* and *CotAD_01947*) had potential involvement in the early maturity response in plants. Two early-maturing cotton varieties and two late-maturing varieties were selected. The WGPs of the early-maturing varieties zhongmiansuo50 and zhongmiansuo74 were 107.92 d and 102.75 d, respectively, and those of the late-maturing varieties lumianyan28 and zhongmiansuo41 were 124.17 d and 126.67 d, respectively (Fig. 8a and b). Similarly, the FT of the early-maturing varieties was significantly shorter than that of the late-maturing varieties (*P* < 0.01) (Fig. 8c). The transcription levels of the 32 genes were assessed by qRT-PCR using samples from the roots, stems, leaves, flowers, ovules and fibers of upland cotton. Examples of these results are shown in Additional file 6: Figure S5A. In particular, high expression levels of *CotAD_01947* and *CotAD_01914* were detected in the leaves, whereas low expression levels were identified in the roots, stems, flowers, ovules and fibers (Fig. 8d and Additional file 6: Figure S5A). In addition, qRT-PCR was used to examine the expression patterns of 16 genes in two early-maturing varieties and two late-maturing cotton varieties at five different leaf growth stages (cotyledon and one-leaf to four-leaf stages). From the two-leaf stage to the four-leaf stage, the expression of *CotAD_01947* in the early-maturing varieties zhongmiansuo50 and zhongmiansuo74 was significantly higher than that in the late-maturing varieties lumianyan28 and zhongmiansuo41 (*P* < 0.01) (Fig. 8e). However, the expression of the other genes investigated did not significantly differ between the early-maturing and late-maturing varieties (Additional file 6: Figure S5B and C). These data provide support for *CotAD_01947* as a candidate gene for early maturity in upland cotton.

**Table 4:** Candidate genes most highly associated with early maturity within 500 kb of either side of the SNP locus *rs13562854*

#GeneID	Start	Stop	Direction	Distance to SNP (kb)	Annotation
*CotAD_01929*	13482736	13483020	Forward	79.83	
*CotAD_01940*	13836983	13837348	Reverse	274.13	Tetratricopeptide repeat-like superfamily protein, putative
*CotAD_01935*	13685536	13686374	Reverse	122.68	Zinc finger protein, putative isoform 1
*CotAD_01920*	13173869	13177058	Forward	385.80	Enolase 1, chloroplastic-like protein
*CotAD_01932*	13669620	13671044	Reverse	106.77	Zinc finger protein, putative isoform 1
*CotAD_01921*	13215464	13215928	Reverse	346.93	Proline and serine-rich 1
*CotAD_01931*	13641931	13643458	Reverse	79.08	Ribonuclease P subunit p30
*CotAD_01934*	13680075	13680812	Forward	117.22	Hypothetical protein F383_23360
*CotAD_01930*	13549901	13550143	Forward	12.71	
*CotAD_01939*	13835211	13836617	Forward	272.36	UDP-glycosyltransferase 89B1-like
*CotAD_01941*	13837426	13838590	Reverse	274.57	Tetratricopeptide repeat-like superfamily protein, putative
*CotAD_01943*	13894122	13897285	Reverse	331.27	Hypothetical protein F383_21541
*CotAD_01949*	14027015	14029396	Reverse	464.16	ADP, ATP carrier protein ER-ANT1-like
*CotAD_01928*	13425578	13427698	Reverse	135.16	DNA-directed RNA polymerases I and III subunit RPAC1
*CotAD_01944*	13922306	13923205	Forward	359.45	
*CotAD_01942*	13839501	13840400	Reverse	276.65	UDP-glucosyl transferase 89B1, putative
*CotAD_01919*	13169627	13172234	Forward	390.62	DnaJ, mitochondrial
*CotAD_01937*	13763420	13764002	Forward	200.57	
*CotAD_01926*	13313301	13314846	Forward	248.01	Zinc finger CONSTANS-LIKE 2-like protein
*CotAD_01915*	13095428	13096529	Forward	466.33	UDP-N-acetylmuramoyl-alanine-D-glutamate ligase
*CotAD_01922*	13233921	13234454	Forward	328.40	
*CotAD_01914*	13066571	13067059	Forward	495.80	Agamous-like MADS-box protein A
*CotAD_01938*	13771588	13773328	Forward	208.73	Crooked neck-like protein 1
*CotAD_01924*	13252910	13256745	Forward	306.11	Serine/threonine protein kinase 16
*CotAD_01946*	13990075	13991007	Forward	427.22	OBF-binding protein 4, putative
*CotAD_01948*	14020947	14021135	Reverse	458.09	Hypothetical protein CISIN_1g035470mg
*CotAD_01947*	14015684	14017498	Reverse	452.83	MADS-box protein
*CotAD_01918*	13135919	13143770	Forward	419.08	Putative acyl-activating enzyme 17, peroxisomal-like protein
*CotAD_01945*	13956974	13957540	Reverse	394.12	ARM repeat superfamily protein
*CotAD_01923*	13236335	13238168	Forward	324.69	Hypothetical protein F383_15236
*CotAD_01916*	13103756	13106200	Forward	456.65	
*CotAD_01936*	13717384	13722424	Reverse	154.53	WD repeat and HMG-box DNA-binding 1

**Fig. 8:**
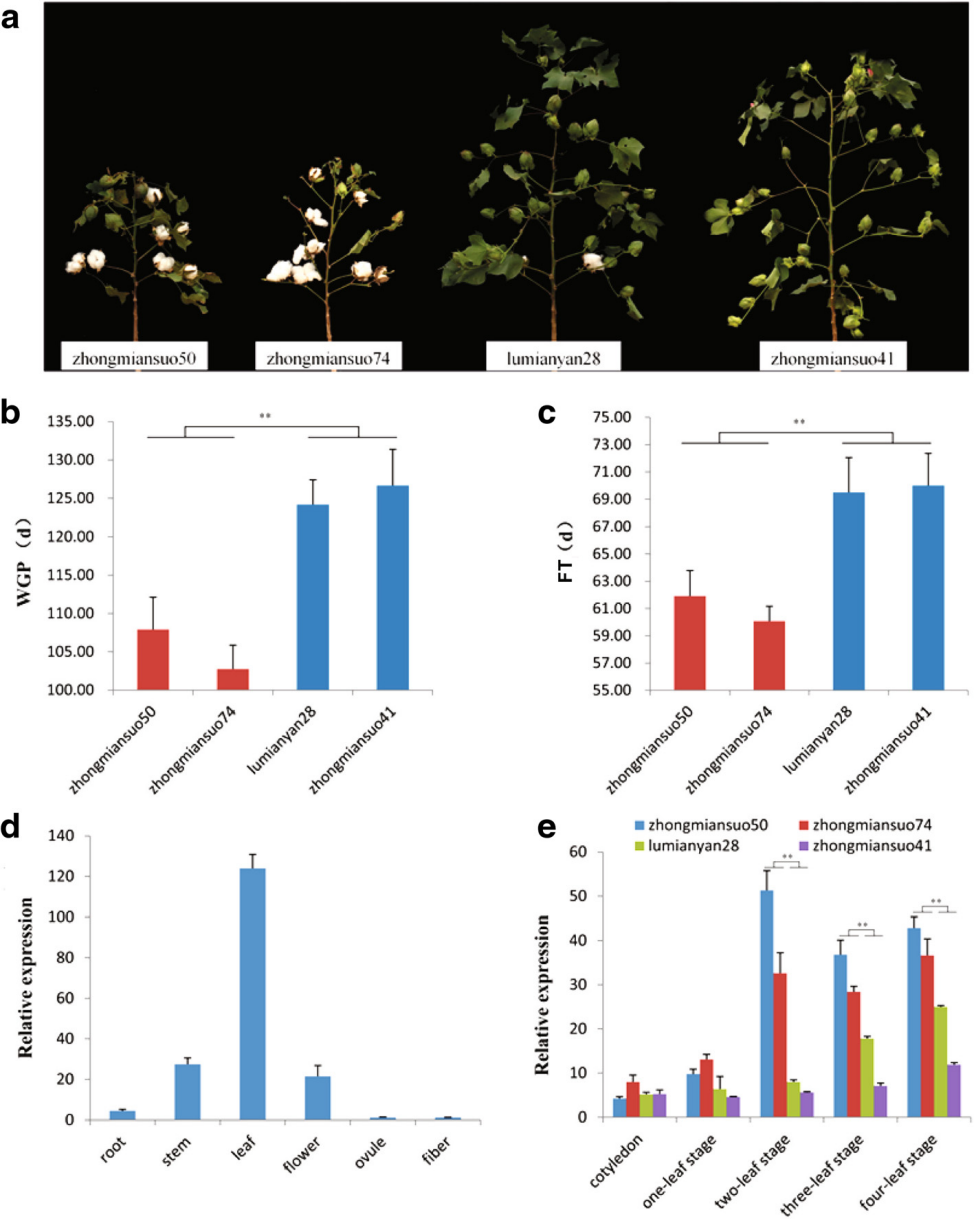
Increased expression of the MADS-box family gene *CotAD_01947* in early-maturing cultivars of upland cotton. **a** Plants at the boll-opening stage of two early-maturing and two late-maturing cotton varieties. **b** and **c** Phenotypic effect values of the WGP and FT for two early-maturing and two late-maturing varieties. **d** Tissue-specific expression patterns of *CotAD_01947*. **e** Expression levels of *CotAD_01947* during the five different leaf growth stages. **indicates significance at the 0.01 probability level

## Discussion

### Identification and verification of SNP loci associated with traits related to early maturity in upland cotton

Both linkage mapping and association analysis provide tools for interpreting the genes that underlie complex traits. To date, linkage mapping is a major method for the mining of QTLs for early maturity traits in cotton. Based on the findings of previous studies, it can be concluded that only preliminary progress has been achieved toward localization of QTLs for cotton early maturity traits with desirable effects in the segregation population (F_2_ populations and recombinant inbred lines (RILs)) [2, 11, 32], and these findings require further verification. Although several studies have identified QTLs for early maturity traits by association analysis in upland cotton [33, 34], these studies were limited by the sizes of the SSR markers and germplasm populations. As the availability of whole-genome sequences has increased and they have become more cost-effective to generate, the practicality of GWAS has increased. In our study, to improve the efficiency and accuracy of association analysis, a wider selection of germplasm resources for upland cotton was collected that was selected based on maturity traits. Further, a substantial number of SNP markers were developed by genome sequencing. Thirteen associations were identified between 8 SNP loci and five early maturity traits (-lg(p) ≥6.21) (Additional file 1: Table S3). Thus, this study has addressed gaps in the study of cotton early maturity traits using GWAS. Most importantly, a main SNP locus for the WGP and FT was identified on chromosome D_t_3.

**Fig. 9:**
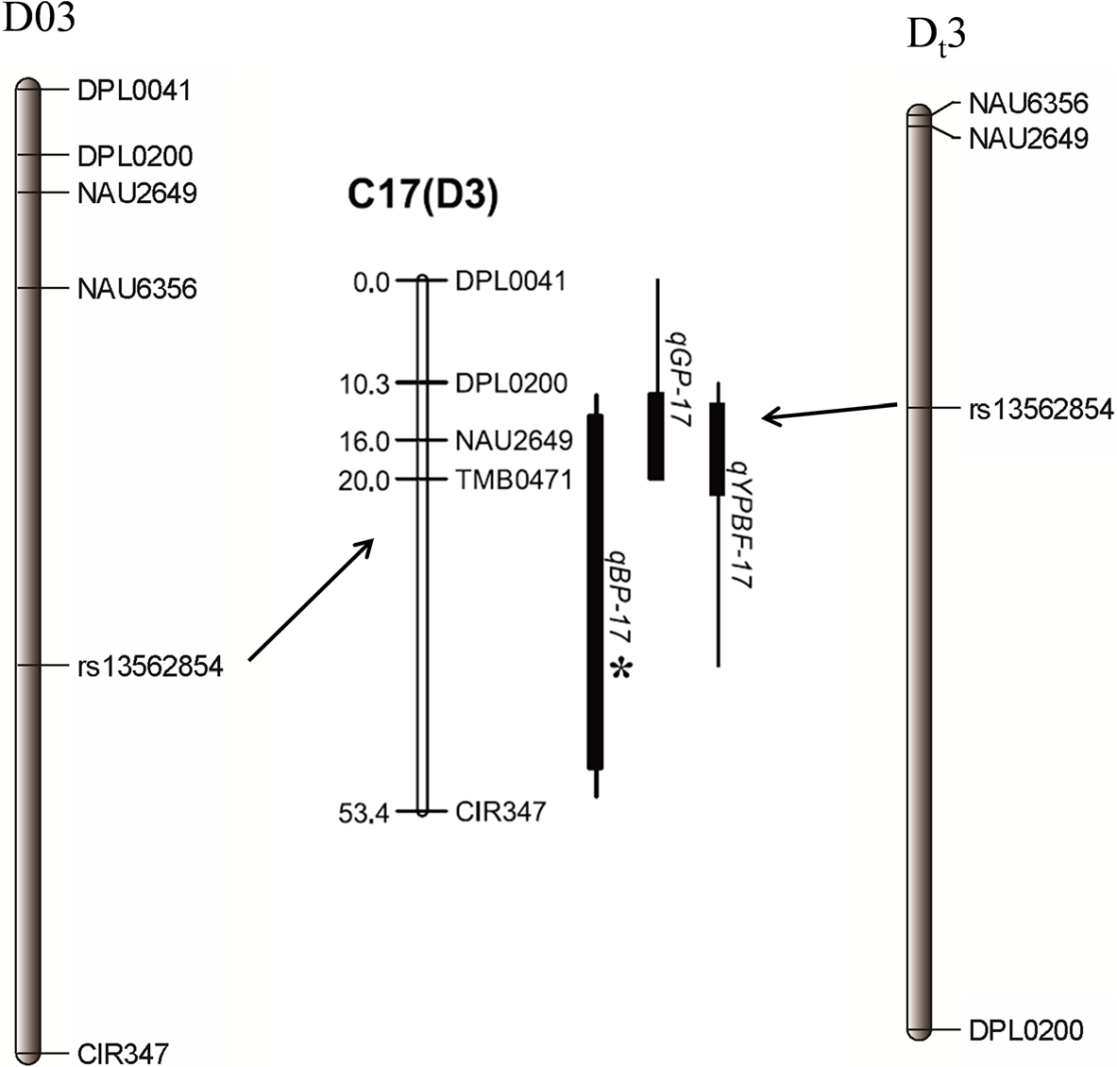
Physical maps and linkage relationships among quantitative trait loci (QTLs) in previous and present studies. Physical maps of the reference *Gossypium hirsutum* genome D03 [56] and D_t_3 [31] from the present study, respectively. Linkage map of C17 (D3) from a previous study [2]

In a previous study, one significant QTL for the GP, BP and YPBF was found to be located close to the bridge markers DPL0041 and CIR347 on Chr17 (D3) in two biparental populations, explaining 20.00 % of the phenotypic variation [2]. The physical locations of these SSR markers were mapped to the genome sequence by electronic PCR (e-PCR) (Fig. 9), and a main SNP locus (*rs13562854*) for the WGP and FT was positioned between DPL0200 and CIR347. This finding validates the GWAS results and increases confidence in the identity of the main SNP locus (*rs13562854*).

### Mining of favorable SNP alleles and candidate genes to improve early maturity in cotton

Obtaining satisfactory yield and quality during a short growing season is complicated due to conflict between early maturity and yield, as well as between early maturity and fiber quality; thus, it is increasingly difficult to simultaneously improve upon these agriculturally desirable traits in early-maturing cotton using traditional breeding methods. Therefore, the mining of favorable SNP (or QTL) alleles is necessary for improving important agronomic traits in upland cotton cultivars via MAS. Association mapping is one of the most effective approaches for the mining of favorable alleles. Elite alleles for fiber-quality traits [30] and yield and its components [28] in upland cotton cultivars/accessions were explored via association analysis. In our study, by comparing the average phenotypic effect value of each allele for the target traits in the thirteen stable associations detected, we identified eleven highly favorable alleles for five early maturity traits (Table 1). Moreover, the examination of favorable SNP alleles and germplasm resources for early maturity traits, such as zhongmiansuo74, xia25, and xiazao3, could be useful for plant breeders; however, the effects of these alleles must be verified. Therefore, the positive effects of highly favorable alleles were selected and assessed. To date, many studies have demonstrated that marker-based gene pyramiding strategies are very effective [35–37]. Dosage pyramiding effects of the highly favorable SNP alleles were also demonstrated (Table 2, Fig. 5); thus, the highly favorable alleles identified in this study have substantial potential for the development of early-maturing upland cotton cultivars in future breeding programs.

Of particular interest, the detailed annotations revealed that the major locus *rs13562854* was located on chromosome D_t_3 and that the 32 candidate genes in the nearby region were the most highly associated with the WGP and FT. Specifically, four candidates (*CotAD_01914*, *CotAD_01926, CotAD_01936* and *CotAD_01947*) related to plant floral development were annotated. *CotAD_01947* and *CotAD_01914* were located -452.83 kb (backward) and 495.80 kb (forward), respectively, from the peak SNP (*rs13562854*), with MADS-box genes that encode transcription factors involved in plant developmental control and signal transduction [38]. Notably, a WD repeat (WDR) gene (*CotAD_01936*) was identified 154.53 kb from the *rs13562854* locus. Plant WDR proteins are intimately involved in various cellular and organismal processes, including cell division and cytokinesis, apoptosis, light signaling and vision, cell motility, flowering, floral development and meristem organization [39]. *CotAD_01947* expression in the early-maturing varieties zhongmiansuo50 and zhongmiansuo74 was significantly higher than that in the late-maturing varieties lumianyan28 and zhongmiansuo41. However, expression of the other genes did not significantly differ between the early-maturing and late-maturing varieties (Additional file 6: Figure S5 B and C).

MADS-box family genes play significant roles in plant growth and development, and they also control flowering time and flower initiation [40, 41]. *AGAMOUS-LIKE8* (*AGL8*, AT5G60910) in *Arabidopsis* is another MADS-box family member that regulates the transcription of genes required for cellular differentiation and floral determination [42–44]. The BLAST alignment results indicated that the coding sequence (CDS) identity of *CotAD_01947* with the *Arabidopsis AGL8* gene was as high as 47.50 % (Additional file 7: Figure S6A) and that *CotAD_01947* encoded a protein that shared 50.90 % sequence identity with the *Arabidopsis* AGL8 protein (Additional file 7: Figure S6B). In addition, although fifty-three MADS-box genes have been identified in upland cotton to date [45], few molecular studies of MADS-box genes in *G. hirsutum* have been conducted. For example, *GhMADS11* affects cell elongation in fibers [46], *GhMADS7* regulates anther development [47], and *GhMADS3* participates in flower development [48]. *GhMADS42* in *Arabidopsis* accelerates flowering, and *GhMADS42* transgenic plants exhibit abnormal floral organ phenotypes [49]. In addition, we found that *CotAD_01947* shared 50.90 % amino acid sequence identity with *Arabidopsis AGL8* (Additional file 7: Figure S6B), that most MADS-box family genes in upland cotton regulated flower development, and that *CotAD_01947* expression in early-maturing cotton was higher than that in late-maturing cotton (Fig. 8e). Thus, it is reasonable to postulate that *CotAD_01947* may be a candidate gene for improving early maturity traits via the regulation and control of early flowering time in upland cotton. However, clear and definite identification of *CotAD_01947* as an annotated MADS-box family gene requires further validation.

## Conclusions

A substantial number of SNP markers in upland cotton were developed through SLAF-seq technology and were used in a GWAS. Thirteen significant associations were identified among eight SNP loci and five traits related to early maturity using the GLM and MLM, and two of the 13 associations were observed in both models. Eleven highly favorable SNP alleles for the WGP, FT, FBP, NFFB and YPBF were identified. Moreover, dosage pyramiding effects of the highly favorable SNP alleles and significant linear correlations between the number of highly favorable alleles and the phenotypic values of target traits were detected. Most importantly, a major locus (*rs13562854*) on chromosome D_t_3 and a potential candidate gene (*CotAD_01947*) for early maturity were detected. The beneficial alleles and candidate gene should be useful for improving early maturity in upland cotton breeding via a molecular design approach.

## Methods

### SLAF-seq, sequencing data analysis and SNP calling

Three hundred fifty-five upland cotton accessions (260 varieties, 71 accessions collected from China, and ten additional varieties, ten accessions introduced from the United States, including the genetic standard line TM-1 and four varieties from central Asia) were used for genome sequencing. Seeds from the 355 upland cotton accessions were obtained from the cotton germplasm collection in our laboratory and from the low-temperature germplasm genebank of the Cotton Research Institute, Chinese Academy of Agricultural Sciences (CRI-CAAS). All accessions had been self-pollinated for more than three generations.

Young leaves of ten plants from each of the 355 varieties/accessions were collected, mixed, frozen in liquid nitrogen, and used for DNA extraction. Genomic DNA was isolated from samples from each cotton variety/accession using the cetyltrimethylammonium bromide (CTAB) method, as described by Paterson et al. [50]; RNase A and proteinase K treatments were used to prevent RNA and protein contamination, and then the DNA extracts were subjected to Illumina sequencing and SSR-PCR amplification.

The SLAF library was constructed as described by Sun et al. [51] with several modifications. A SLAF pilot experiment was performed, and the SLAF library was generated in accordance with the predesigned scheme. For this population, two enzymes (*RsaI* and *HaeIII*, New England Biolabs, NEB, USA) were used to digest the genomic DNA. A single nucleotide (A) overhang was subsequently added to the digested fragments using Klenow Fragment (3′ → 5′ exo^−^) (NEB) and dATP at 37 °C. Duplex tag-labeled sequencing adapters (PAGE-purified, Life Technologies, USA) were then ligated to the A-tailed fragments using T4 DNA ligase. PCR was performed using diluted restriction-ligation DNA samples, dNTP, Q5® High-Fidelity DNA Polymerase and PCR primers (forward primer: 5′-AATGATACGGCGACCACCGA-3′; and reverse primer: 5′-CAAGCAGAAGACGGCATACG-3′) (PAGE-purified, Life Technologies). Next, the PCR products were purified using Agencourt AMPure XP beads (Beckman Coulter, High Wycombe, UK) and pooled. The pooled samples were separated by 2 % agarose gel electrophoresis. Fragments that ranged in size from 314 to 364 bp (with indexes and adaptors) were excised and purified using a QIAquick gel extraction kit (Qiagen, Hilden, Germany). The gel-purified products were subsequently diluted. Paired-end sequencing (125 bp at each end) was performed using an Illumina HiSeq 2500 system (Illumina, Inc.; San Diego, CA, USA) according to the manufacturer’s recommendations.

The raw reads (100 bp in length) were filtered and trimmed as follows: reads with ≥10 % unknown nucleotides were removed; reads with ≥30 % low-quality bases (base quality ≤10) were removed; reads with clear index information were trimmed; and low-quality bases at the 3′ ends of reads were removed. Read quality was considered acceptable if the Q30 ratio was ≥80 % after trimming and a paired sequence length of 80 bp was retained at each end. To evaluate sequence quality, real-time monitoring was performed in each cycle during sequencing, and the ratio of the number of high-quality reads with quality scores > Q30 (a quality score of 30 indicates a 0.10 % chance of an error and thus 99.90 % confidence) to the total number of raw reads and the GC content were calculated. BWA software was used to map the raw paired-end reads to the reference genome (*Gossypium hirsutum* v 1.0) [31]. SLAF groups were generated by grouping reads that were mapped to the same position. If an accession was only partly digested by the restriction enzymes, some reads that mapped to the reference genome overlapped by two SLAF tags. These reads were assigned to both SLAF tags in the accession. The GATK and SAMtools packages were used for SNP calling.

### Population structure and linkage disequilibrium estimation

The ADMIXTURE [52] program was used to assess the population structure based on the maximum-likelihood method with 10,000 iterations, and the number of clusters (K) was set from 2 to 10. The SNPs were used after filtering for an MAF >0.05 and an identity of greater than 80 %. Pairwise LD between markers was calculated as the squared correlation coefficient (r^2^) of alleles using GAPIT software [53].

### Field experiments and collection and analysis of phenotypic data

A subset of 185 lines was selected from the 355 upland cotton accessions from the cotton germplasm collection in our laboratory and from the low-temperature germplasm genebank of the CRI-CAAS. Selection was based on analyses of population structure and maturity, with the genotypes from the nine subpopulations characterized into two main groups according to maturity traits. The first group (103 genotypes) contained the early-maturing genotypes, including 76 varieties/accessions that originated from the Yellow River region, 15 varieties/accessions that originated from the northern specific early-maturing region, ten varieties/accessions that originated from the northwestern inland early-maturing region and two varieties introduced from the United States. The second group (82 genotypes) contained the late-maturing genotypes, including 69 varieties/accessions that originated from the Yellow River region, five varieties/accessions that originated from the Yangtze River region and 8 varieties introduced from the United States (Additional file 1: Table S4).

The population was planted at the experimental station of the CRI-CAAS in Anyang, Henan (36°05 N; 114°21E). All cotton lines were sown at two time points, including late April and late May (referred to as SP-sowing and SU-sowing, respectively), in 2013 and 2014. The different cotton varieties/accessions were each grown in a single-row plot (5.0 m long and 0.8 m row wide), with three replicates and a random complete block design. The field management conformed to local practices.

The following six traits related to early maturity were investigated in this study: WGP (the period from sowing to the first boll opening), FT (the period from sowing to the first flower blooming), FBP (the period from the first flower blooming to the first boll opening), NFFB (the number of nodes from the cotyledon node to the first fruiting branch node), and HNFFB (the distance between the cotyledon node and the NFFB) and YPBF (the seed yield percentage before October 25th). Ten consecutive plants in the middle of each row were tagged for trait measurements. These plants were observed, and the average value of three replicates was recorded. The phenotypic data were analyzed using SAS 9.3 statistical software (SAS, Chicago, IL, USA). To reduce environmental error, BLUPs for six early maturity traits per genotype were obtained using the PROC MIXED procedure of SAS9.3. ANOVA was performed using PROC ANOVA. Linear regression analysis was conducted using the GLM procedure in SAS.

### GWAS and favorable allele identification

For all SNP loci and phenotypic data, we applied the GLM and MLM. In addition, to minimize the effects of environmental variation, BLUPs were computed for GWAS [24]. The BLUP values for the four environments and the phenotypic values of six early maturity traits for each environment were used in GWAS. The high-quality SNPs were filtered according to the MAF (MAF >0.05) and the integrity of each SNP (>50 %). These SNPs from 185 cotton accessions were used in association analysis conducted using the GLM and MLM with GAPIT software [53]. Bonferroni-adjusted *P*-values of ≤0.01 and 0.05 (-lg(p) ≥ 6.91 and -lg(p) ≥ 6.21, respectively) were used as thresholds to determine whether significant associations existed [54]. SNP loci significantly associated with the target traits based on the GWAS results were analyzed. According to the computational method described by Zhang et al. [29], the phenotypic effect of each SNP locus (a_i_) was estimated through comparison of the average phenotypic value for each accession for the specific locus with that of all accessions. The favorable alleles were subsequently identified according to the breeding objective of each target trait. For the WGP, FT, FBP, NFFB and HNFFB, a_i_ < 0 indicates a favorable allele, and for the YPBF, a_i_ > 0 indicates a favorable allele.

### Quantitative real-time PCR

Total RNA was isolated from the samples using a Plant RNA Purification Kit (Tiangen, Beijing, China). Reverse transcription was conducted using a SuperScript III First-Stand Synthesis System to obtain cDNA for qRT-PCR (Invitrogen, Carlsbad, CA, USA). Transcript levels were subsequently determined by qRT-PCR using a 7500 Real-Time PCR System (Applied Biosystems, Foster City, CA, USA) and SYBR PremixEx Taq (2×) (TaKaRa). The gene-specific primer pairs used for PCR amplification are listed in Additional file 1: Table S5 and were designed to avoid conserved regions. To normalize the variance among samples, *actin* was used as an endogenous control, and the gene expression levels were calculated using the 2^−ΔΔCT^ method [55].

## Abbreviations

ANOVA, analysis of variance; BLUP, best linear unbiased prediction; CV, coefficients of variance; FBP, flowering and boll-setting period; FT, flowering time; GLM, generalized linear model; GWAS, genome-wide association study; HNFFB, height of the node of the first fruiting branch; LD, linkage disequilibrium; MAF, minor allele frequency; MAS, marker-assisted selection; MLM, mixed linear model; NFFB, node of the first fruiting branch; SLAF-seq, specific-locus amplified fragment sequencing; SNP, single nucleotide polymorphism; SSR, simple sequence repeat; WGP, whole growth period; YPBF, yield percentage before frost

## Additional files

Additional file 1: Table S1. Descriptive statistics for six traits related to early maturity in four different environments. **Table S2.** Correlations between the WGPs and related traits based on 185 upland cotton accessions in four environments. **Table S3.** Single nucleotide polymorphisms (SNPs) significantly associated with traits related to early maturity in upland cotton, as determined using the GLM and MLM. **Table S4.** Information on 185 upland cotton germplasms. **Table S5.** qRT-PCR primers. (DOCX 45 kb) Additional file 2: Figure S1. Manhattan plots of genome-wide association studies (GWAS) for the WGP measured with the GLM using the phenotypic values for the different environments. (JPG 4500 kb) Additional file 3: Figure S2. Manhattan plots of genome-wide association studies (GWAS) for the FT measured with the GLM using the phenotypic values for the different environments. (JPG 7388 kb) Additional file 4: Figure S3. Manhattan plots of genome-wide association studies (GWAS) for the WGP measured with the MLM using the phenotypic values for the different environments. (JPG 7101 kb) Additional file 5: Figure S4. Manhattan plots of genome-wide association studies (GWAS) for the FT measured with the MLM using the phenotypic values for the different environments. (JPG 7017 kb) Additional file 6: Figure S5. Expression levels of several candidate genes that potentially underlie early maturity. (JPG 3083 kb) Additional file 7: Figure S6. Structure and identity of the MADS-box gene homolog *CotAD_01947*. (JPG 1502 kb)
