# Supplementary material for: Identification of favorable SNP alleles and candidate genes for traits related to early maturity via GWAS in upland cotton
Source: BMC Genomics. 2016 Aug 30;17(1):687. doi: 10.1186/s12864-016-2875-z (PMC5006539; doi:10.1186/s12864-016-2875-z)
Supplement: Additional file 1: Table S1. — Descriptive statistics for six traits related to early maturity in four different environments. Table S2. Correlations between the WGPs and related traits based on 185 upland cotton accessions in four environments. Table S3. Single nucleotide polymorphisms (SNPs) significantly associated with traits related to early maturity in upland cotton, as determined using the GLM and MLM. Table S4. Information on 185 upland cotton germplasms. Table S5. qRT-PCR primers. (DOCX 45 kb) [file 12864_2016_2875_MOESM1_ESM.docx]

Table S1 Descriptive statistics for six traits related to early maturity in four different environments

| Traits^a^ | Environments^b^ | Mean | SD | Min | Max | CV (%) | G | G×E |
| --- | --- | --- | --- | --- | --- | --- | --- | --- |
| WGP (d) | SP-2013 | 116.61 | 5.98 | 102.33 | 129.00 | 5.13 | ** | ** |
|  | SU-2013 | 117.92 | 13.91 | 96.67 | 141.33 | 11.80 |  |  |
|  | SP-2014 | 118.03 | 8.98 | 102.33 | 147.00 | 7.61 |  |  |
|  | SU-2014 | 120.39 | 7.86 | 103.67 | 138.00 | 6.53 |  |  |
|  | Mean | 118.24 | 8.14 | 102.75 | 135.83 | 6.88 |  |  |
| FT (d) | SP-2013 | 69.75 | 3.18 | 63.33 | 77.67 | 4.56 | ** | ** |
|  | SU-2013 | 68.75 | 8.59 | 53.00 | 80.67 | 12.49 |  |  |
|  | SP-2014 | 65.81 | 4.11 | 60.67 | 80.67 | 6.25 |  |  |
|  | SU-2014 | 62.05 | 2.15 | 58.33 | 68.33 | 3.46 |  |  |
|  | Mean | 66.59 | 3.93 | 59.50 | 75.25 | 5.91 |  |  |
| FBP (d) | SP-2013 | 46.86 | 3.46 | 38.00 | 54.00 | 7.38 | ** | ** |
|  | SU-2013 | 49.13 | 6.01 | 37.00 | 61.00 | 12.23 |  |  |
|  | SP-2014 | 52.21 | 5.78 | 41.33 | 73.33 | 11.07 |  |  |
|  | SU-2014 | 58.35 | 6.40 | 44.33 | 73.67 | 10.97 |  |  |
|  | Mean | 51.64 | 4.54 | 42.42 | 63.83 | 8.79 |  |  |
| NFFB | SP-2013 | 6.48 | 1.26 | 3.00 | 12.00 | 19.44 | ** | ** |
|  | SU-2013 | 7.62 | 1.58 | 4.21 | 11.71 | 20.73 |  |  |
|  | SP-2014 | 5.97 | 0.73 | 4.57 | 7.80 | 12.23 |  |  |
|  | SU-2014 | 5.93 | 0.82 | 4.00 | 7.60 | 13.83 |  |  |
|  | Mean | 6.50 | 1.03 | 4.34 | 8.95 | 15.79 |  |  |
| HNFFB (cm) | SP-2013 | 23.61 | 3.24 | 15.81 | 36.10 | 13.72 | ** | ** |
|  | SU-2013 | 28.87 | 7.18 | 16.67 | 47.54 | 24.87 |  |  |
|  | SP-2014 | 18.05 | 3.25 | 11.20 | 26.20 | 18.01 |  |  |
|  | SU-2014 | 22.45 | 3.83 | 15.03 | 31.27 | 17.06 |  |  |
|  | Mean | 23.25 | 3.93 | 15.45 | 34.03 | 16.92 |  |  |
| YPBF (%) | SP-2013 | 79.43 | 13.48 | 28.03 | 97.54 | 16.97 | ** | ** |
|  | SU-2013 | 47.58 | 28.45 | 1.55 | 95.47 | 59.79 |  |  |
|  | SP-2014 | 85.07 | 6.81 | 54.58 | 100.00 | 8.01 |  |  |
|  | SU-2014 | 67.80 | 8.79 | 40.88 | 79.42 | 12.96 |  |  |
|  | Mean | 69.97 | 12.67 | 39.89 | 90.51 | 18.11 |  |  |

^a^ WGP: whole growth period (d); FT: flowering time (d); FBP: flowering and boll-setting period (d); NFFB: node of first fruiting branch; HNFFB: height of node of first fruiting branch (cm); YPBF: yield percentage before frost (%).

^b^ SP-2013, SU-2013, SP-2014 and SU-2014: the different planting environments of two sowing stages (spring, SP; summer, SU) in 2013 and 2014.

** indicates significance at the 0.01 probability level.

Table S2 Correlations among the WGPs and the [related](javascript:void(0);) traits based on the 185 upland cotton accessions in four environments.

| Environments | FBP | FT | NFFB | HNFFB | YPBF |
| --- | --- | --- | --- | --- | --- |
| WGP (SP-2013) | 0.9079^**^ | 0.8899^**^ | 0.8461^**^ | 0.5740^**^ | -0.7640^**^ |
| WGP (SU-2013) | 0.9306^**^ | 0.9690^**^ | 0.8946^**^ | 0.8824^**^ | -0.9518^**^ |
| WGP (SP-2014) | 0.9363^**^ | 0.8697^**^ | 0.5489^**^ | 0.5407^**^ | -0.6091^**^ |
| WGP (SU-2014) | 0.9754^**^ | 0.7538^**^ | 0.7156^**^ | 0.7511^**^ | -0.6875^**^ |
| Mean | 0.9541^**^ | 0.9659^**^ | 0.8775^**^ | 0.8513^**^ | -0.9230^**^ |

^**^ significantly different (P < 0.01).

Table S3 Single nucleotide polymorphisms (SNPs) significantly associated with traits related to early maturity in upland cotton, as determined using the GLM and MLM

|  | Traits | SNP | Position^a^ | BULP |  | SP-2013 |  | SP-2014 |  | SU-2013 |  | SU-2014 |  |
| --- | --- | --- | --- | --- | --- | --- | --- | --- | --- | --- | --- | --- | --- |
|  |  |  |  | -lg(p) | R^2^ (%) | -lg(p) | R^2^ (%) | -lg(p) | R^2^ (%) | -lg(p) | R^2^ (%) | -lg(p) | R^2^ (%) |
| GLM | WGP | *rs26538646* | A_t_3:26538646 | 9.50 | 8.00 | 6.25 | 5.77 | 7.77 | 13.60 | 8.08 | 5.65 |  |  |
|  |  | *rs26538688* | A_t_3:26538688 | 10.03 | 8.39 | 6.43 | 6.02 | 8.17 | 14.21 | 8.29 | 5.77 |  |  |
|  |  | *rs8917898* | D_t_3:8917898 | 8.81 | 8.09 | 6.30 | 6.10 | 8.55 | 13.95 |  |  |  |  |
|  |  | *rs13153008* | D_t_3:13153008 | 9.76 | 8.08 | 7.39 | 6.94 | 7.30 | 11.30 | 6.71 | 4.61 |  |  |
|  |  | *rs13562854* | D_t_3:13562854 | 10.95 | 9.75 | 9.56 | 9.17 | 6.32 | 10.60 | 10.31 | 7.97 |  |  |
|  | FT | *rs22465987* | A_t_4:22465987 | 7.11 | 4.63 | 6.50 | 5.36 | 6.45 | 8.98 |  |  |  |  |
|  |  | *rs48627288* | A_t_12:48627288 | 8.25 | 7.82 | 7.96 | 8.53 | 6.91 | 10.62 |  |  |  |  |
|  |  | *rs8917898* | D_t_3:8917898 | 9.65 | 8.58 | 6.64 | 7.62 |  |  | 6.76 | 5.47 |  |  |
|  |  | *rs13562854* | D_t_3:13562854 | 13.09 | 11.09 | 7.29 | 8.27 |  |  | 15.56 | 11.27 |  |  |
|  |  | *rs37255056* | D_t_3:37255056 | 7.62 | 6.85 | 6.21 | 7.18 | 6.17 | 10.06 |  |  |  |  |
|  | FBP | *rs13153008* | D_t_3:13153008 | 8.88 | 9.34 | 6.95 | 8.96 | 6.53 | 11.73 |  |  |  |  |
|  | NFFB | *rs13562854* | D_t_3:13562854 | 8.26 | 7.77 | 6.94 | 7.81 |  |  | 8.21 | 8.19 |  |  |
|  | YPBF | *rs13562854* | D_t_3:13562854 | 10.62 | 10.53 |  |  |  |  | 12.29 | 9.73 | 6.48 | 9.36 |
| MLM | WGP | *rs13562854* | D_t_3:13562854 | 6.25 | 10.54 | 6.22 | 9.23 |  |  | 6.56 | 11.21 |  |  |
|  | FT | *rs13562854* | D_t_3:13562854 | 7.01 | 14.09 |  |  | 6.22 | 10.24 | 8.11 | 16.46 |  |  |

^a^: Chromosome position according to Li et al (2015); GLM: generalized linear model; MLM: mixed linear model; BLUP: best linear unbiased predictions; R^2^: variation explained by the SNPs; p is the statistical p-value for the significance of the odds ratio in the GWAS.

Table S4 A subset of 185 lines was selected from the 355 upland cotton accessions

| No. | accessions | region | No. | accessions | region | No. | accessions | region | No. | accessions | region | No. | accessions | region |
| --- | --- | --- | --- | --- | --- | --- | --- | --- | --- | --- | --- | --- | --- | --- |
| 1 | zhong02191 | A | 21 | 29-41 | A | 41 | heishanmian1 | D | 61 | shizao3 | A | 81 | yunzaoN177 | A |
| 2 | zhong040418 | A | 22 | 29-42 | A | 42 | jinmian3 | D | 62 | xia13-7 | A | 82 | yunzaoN95 | A |
| 3 | zhong040618 | A | 23 | 6426 | A | 43 | jinmian10 | A | 63 | xia25 | A | 83 | zhong416 | A |
| 4 | zhong040712 | A | 24 | K640 | A | 44 | jinmian21 | A | 64 | xiazao1 | A | 84 | zhong425-5 | A |
| 5 | zhong051811 | A | 25 | N82 | A | 45 | jinmian23 | A | 65 | xiazao2 | A | 85 | zhong716 | A |
| 6 | zhong051822 | A | 26 | P21-6-7 | B | 46 | jinmian5 | A | 66 | xiazao3 | A | 86 | zhong751213 | A |
| 7 | zhong061832 | A | 27 | SF06 | A | 47 | liaomian10 | D | 67 | xinluzao11 | C | 87 | zhongchuang88 | A |
| 8 | zhong071239 | A | 28 | SGK16 | B | 48 | liaomian17 | D | 68 | xinluzao36 | C | 88 | zhongmiansuo10 | A |
| 9 | PB12-1-10 | A | 29 | V321-20-14 | B | 49 | liaomian5 | D | 69 | xinluzao3 | C | 89 | zhongmiansuo14 | A |
| 10 | PB12-1-7 | A | 30 | baimian17 | A | 50 | liaomian6 | D | 70 | xinluzao42 | C | 90 | zhongmiansuo16 | A |
| 11 | PB12-1-8 | A | 31 | chaoyangmian1 | D | 51 | liaomian7 | D | 71 | xinluzao45 | C | 91 | zhongmiansuo20 | A |
| 12 | zhong1476 | A | 32 | daizimian20 | E | 52 | liaomian9 | D | 72 | xinluzao4 | C | 92 | zhongmiansuo24 | A |
| 13 | zhong151222 | A | 33 | de97-047 | A | 53 | liaoyangduanjie | D | 73 | xinluzao6 | C | 93 | zhongmiansuo27 | A |
| 14 | zhong152201 | A | 34 | guannong1 | D | 54 | lu154 | A | 74 | xinluzao8 | C | 94 | zhongmiansuo30 | A |
| 15 | SQ152201 | A | 35 | han2490 | A | 55 | lu890 | A | 75 | xinluzao9 | C | 95 | zhongmiansuo36 | A |
| 16 | zhong152214 | A | 36 | han656 | A | 56 | lumina2153 | A | 76 | xinxiang368 | A | 96 | han256 | A |
| 17 | SQ152224 | A | 37 | han559 | A | 57 | nongken5 | C | 77 | yu1335 | A | 97 | zhongmiansuo37 | A |
| 18 | 2011SS | A | 38 | han667 | A | 58 | shan70 | A | 78 | yumian12 | A | 98 | zhongmiansuo42 | A |
| 19 | QS2012-3 | A | 39 | han686 | A | 59 | shizao1 | A | 79 | yuzao8E13 | A | 99 | zhongmiansuo50 | A |
| 20 | QS2012-4 | A | 40 | han9609 | A | 60 | shizao2 | A | 80 | yuzao910 | A | 100 | zhongmiansuo58 | A |

| No. | accessions | region | No. | accessions | region | No. | accessions | region | No. | accessions | region | No. | accessions | region |
| --- | --- | --- | --- | --- | --- | --- | --- | --- | --- | --- | --- | --- | --- | --- |
| 101 | zhongmiansuo64 | A | 118 | SPPB12-1-9 | A | 135 | cang198 | A | 152 | lu7619 | A | 169 | zhongmiansuo17 | A |
| 102 | zhongmiansuo74 | A | 119 | SPPB12-2-7 | A | 136 | chunbeibao | A | 153 | lumianyan17 | A | 170 | zhongmiansuo19 | A |
| 103 | 000776 | A | 120 | 298 | A | 137 | chunnanbao | A | 154 | lumianyan21 | A | 171 | zhongmiansuo43 | A |
| 104 | 020398 | A | 121 | 602186 | A | 138 | daizimian14 | E | 155 | lumianyan28 | A | 172 | zhongmiansuo60 | A |
| 105 | 061930 | A | 122 | CG3020-1 | E | 139 | daizimian15 | E | 156 | lumianyan36 | A | 173 | zhong662 | A |
| 106 | 061995 | A | 123 | CG3020-3 | E | 140 | fanmian3 | A | 157 | lumianyan38 | A | 174 | zhong679 | A |
| 107 | 102909 | A | 124 | FM1735 | E | 141 | ganzao109 | B | 158 | miaobao21 | A | 175 | zhongmiansuo69 | A |
| 108 | 103026 | A | 125 | G2005 | A | 142 | guoxinmian11 | A | 159 | renhe39 | A | 176 | zhong800319 | A |
| 109 | 103028 | A | 126 | GK44 | B | 143 | han7860 | A | 160 | rihuimian6 | A | 177 | zhong915 | A |
| 110 | 103030 | A | 127 | H109 | A | 144 | ji4025 | A | 161 | shannongSF01 | A | 178 | zhongmiansuo12 | A |
| 111 | 103032 | A | 128 | H559 | A | 145 | jinmian26 | A | 162 | shan79 | A | 179 | zhongmiansuo35 | A |
| 112 | 103075 | A | 129 | LIH33 | A | 146 | BM03 | A | 163 | sizimian2B | E | 180 | zhongmiansuo41 | A |
| 113 | 103164 | A | 130 | Phy-7 | E | 147 | kelin098 | A | 164 | xinmian33B | A | 181 | zhongmiansuo45 | A |
| 114 | 103245 | A | 131 | STS458 | E | 148 | liaomian23 | D | 165 | xinzhimian5 | A | 182 | zhongmiansuo49 | A |
| 115 | 103297 | A | 132 | TM-1 | E | 149 | liaomian27 | D | 166 | yinhuashu | A | 183 | zhongmiansuo7 | A |
| 116 | 109056 | A | 133 | aoshimian4406 | A | 150 | liaomian28 | D | 167 | you009 | A | 184 | zhongzhimian8 | A |
| 117 | SPPB12-1-7 | A | 134 | baimian985 | A | 151 | lu05R59 | D | 168 | zhong109 | A | 185 | zhongzhimianGD89 | A |

A: varieties/accessions coming from Yellow River region ; B: varieties/accessions coming from Yangtze River region; C: varieties/accessions coming from north western inland early-maturity region; D: varieties/accessions coming from northern specific early-maturity region ; E: varieties introduced from the United States.

Table S5: qRT-PCR primers

| Primer | Sequence (5’~ 3’) | Primer | Sequence (5’~ 3’) |
| --- | --- | --- | --- |
| CotAD_01914-F | GACGATGGTAATGCCCAAAGC | CotAD_01914-R | AACATCATCCAATCGCCTTCC |
| CotAD_01915-F | ACTGCTAAGGGAGATAGGACTGG | CotAD_01915-R | CCTTGAGTTTGTAGTGGGGTAGA |
| CotAD_01919-F | AAGCGGCTCGGCATTAGT | CotAD_01919-R | ATGGGAGGAAGTTCGACAAAA |
| CotAD_01920-F | ATCATTGACAGCAGGGGTAATC | CotAD_01920-R | ACCTTTGCCGCCATAGACA |
| CotAD_01921-F | TCCGAGTCTTATGGCAGGTTG | CotAD_01921-R | CGGTAGGGGTTGTGGTTGAT |
| CotAD_01924-F | TATTGATGGATTTTGGGAGTGC | CotAD_01924-R | AGGGGCTTGGAAAGGTGCT |
| CotAD_01926-F | TGACTCCGCTTACCTCTGCC | CotAD_01926-R | ACCATCCTGTTGAACGCTTG |
| CotAD_01928-F | CCACCGCATCTTGAACTCC | CotAD_01928-R | TCACTCCCATTAGGCAACCA |
| CotAD_01929-F | ATTCTCCACTTCCCTATCTATTCG | CotAD_01929-R | GGATGGTTTCGTTGTTCTTTTC |
| CotAD_01931-F | AGGAGGTTATCAGAGTTGAAGCAG | CotAD_01931-R | AGCATATTACAGTGGGTAGGGTG |
| CotAD_01936-F | TGGTACATGCGGTTTGAAGG | CotAD_01936-R | ACATGGGTGACGATGGCTAA |
| CotAD_01938-F | AGCAGTTATTCGTTGCATTCG | CotAD_01938-R | TCCTCCCCTTATTGCCTACAC |
| CotAD_01939-F | TGCTCCAATGGTTTCAGTCC | CotAD_01939-R | GAGTTGATTTGATAGGCAGGGTA |
| CotAD_01945-F | TGCTCCAATGGTTTCAGTCC | CotAD_01945-R | GAGTTGATTTGATAGGCAGGGTA |
| CotAD_01947-F | GGAGAAAACTAATGTGGAGCAGG | CotAD_01947-R | TCAAGGTGGTGGCGAATCAT |
| CotAD_01949-F | GAGGGGTAACCAAGCCAATG | CotAD_01949-R | ATTTCCAGCAAACCACATCG |
