# Supplementary figures and images for: Identification of favorable SNP alleles and candidate genes for traits related to early maturity via GWAS in upland cotton
Source: BMC Genomics. 2016 Aug 30;17(1):687. doi: 10.1186/s12864-016-2875-z (PMC5006539; doi:10.1186/s12864-016-2875-z)

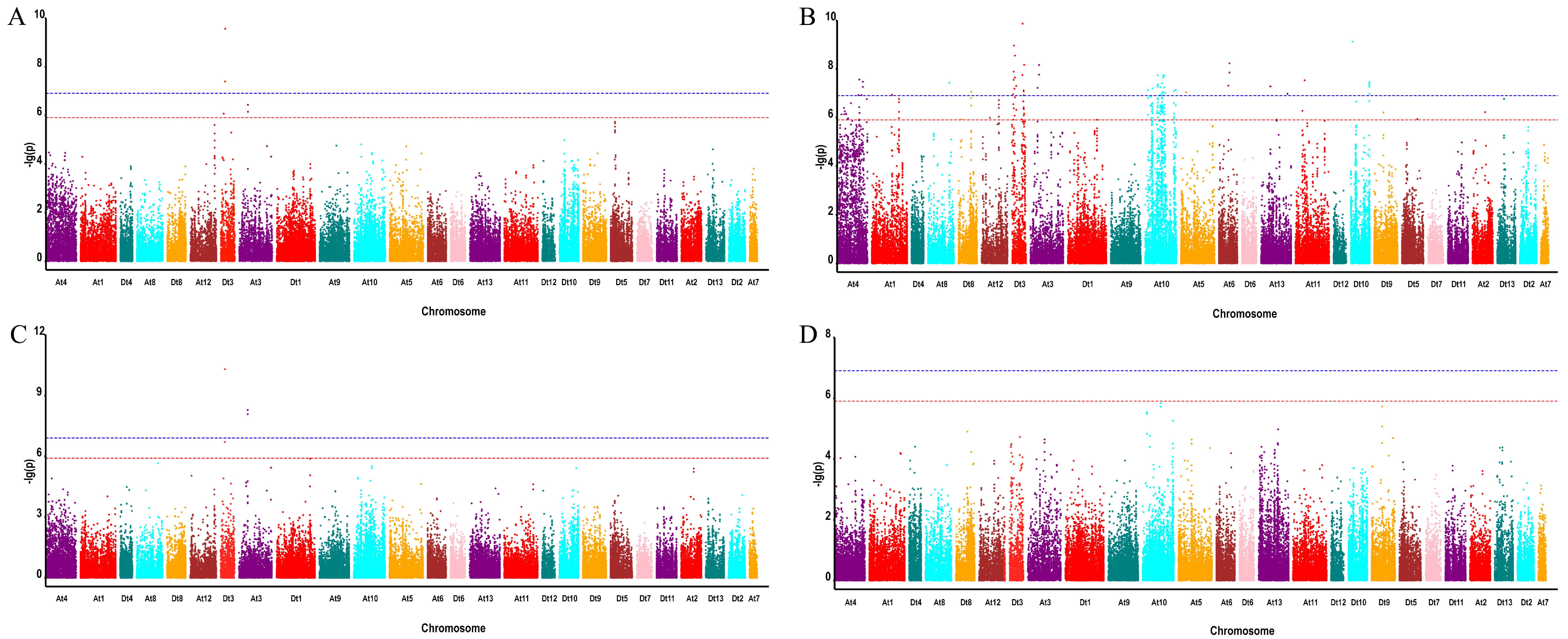

Supplement: Additional file 2: Figure S1. — Manhattan plots of genome-wide association studies (GWAS) for the WGP measured with the GLM using the phenotypic values for the different environments. (JPG 4500 kb) [file 12864_2016_2875_MOESM2_ESM.jpg]

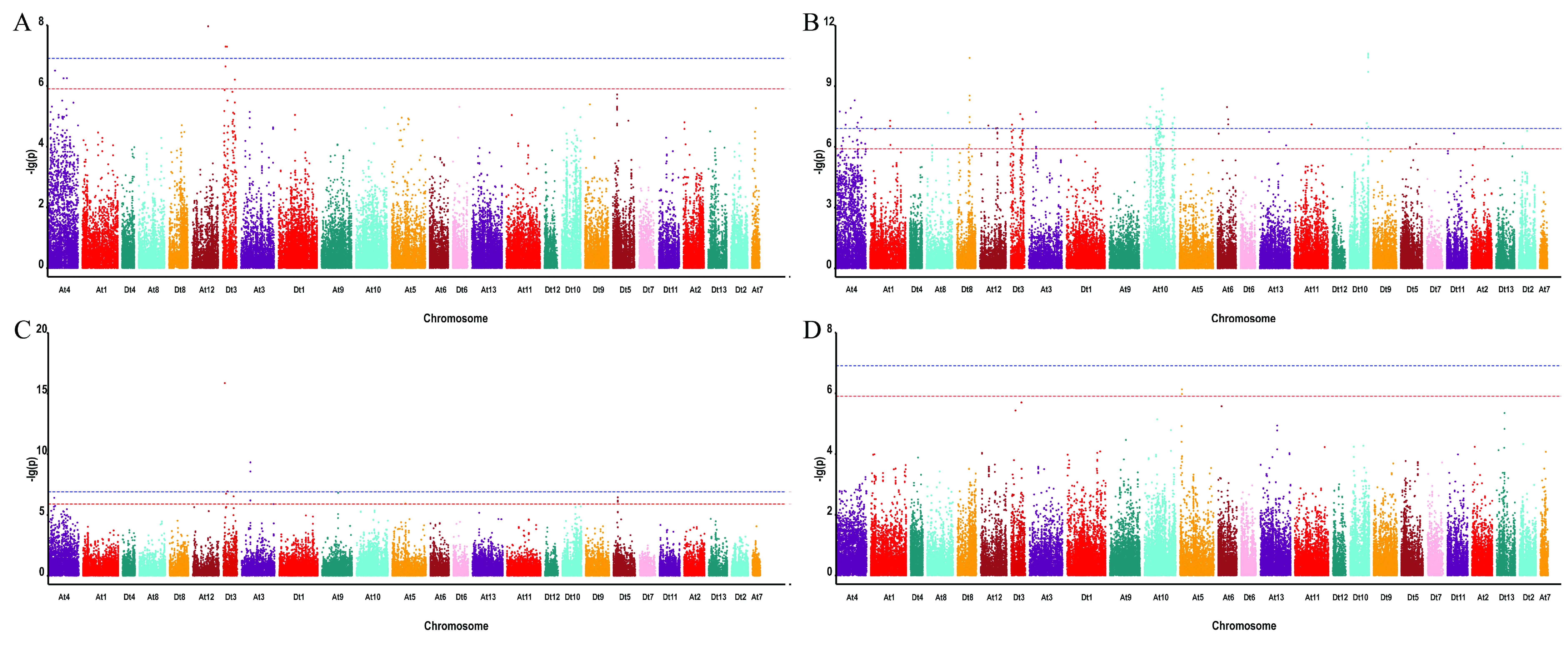

Supplement: Additional file 3: Figure S2. — Manhattan plots of genome-wide association studies (GWAS) for the FT measured with the GLM using the phenotypic values for the different environments. (JPG 7388 kb) [file 12864_2016_2875_MOESM3_ESM.jpg]

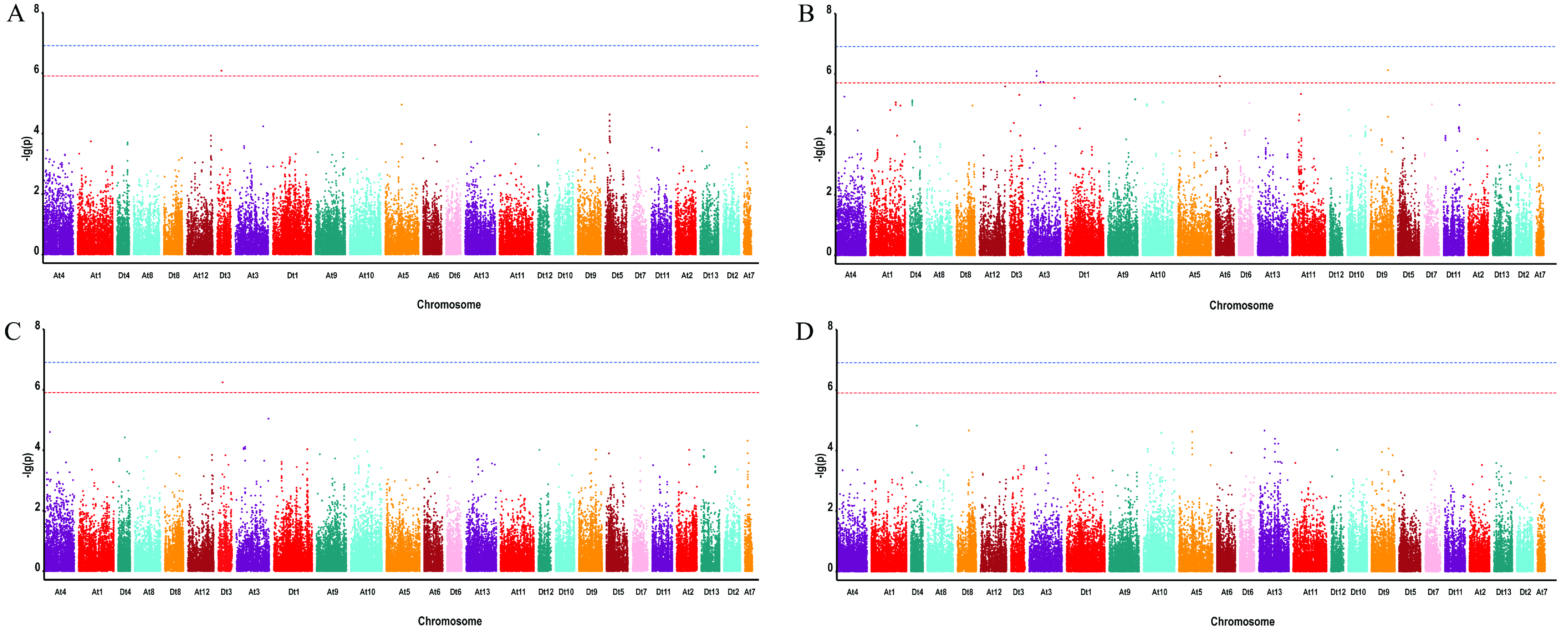

Supplement: Additional file 4: Figure S3. — Manhattan plots of genome-wide association studies (GWAS) for the WGP measured with the MLM using the phenotypic values for the different environments. (JPG 7101 kb) [file 12864_2016_2875_MOESM4_ESM.jpg]

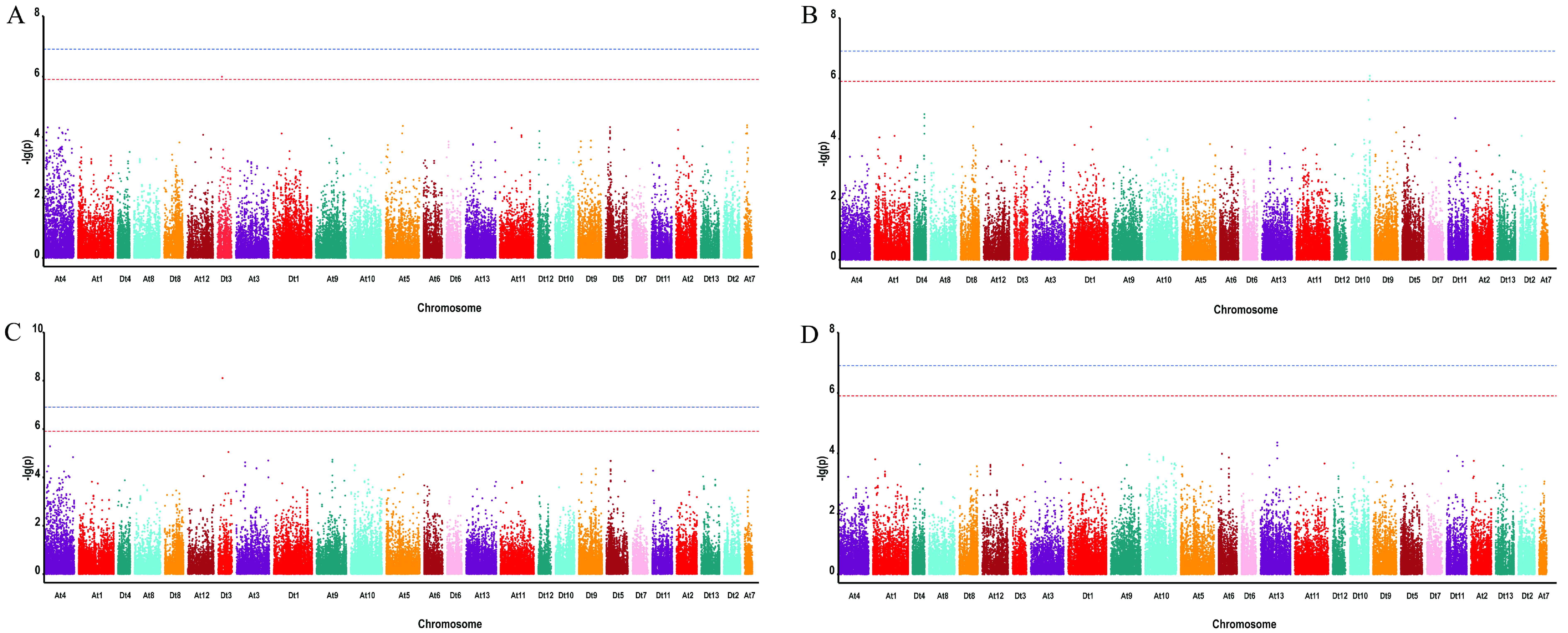

Supplement: Additional file 5: Figure S4. — Manhattan plots of genome-wide association studies (GWAS) for the FT measured with the MLM using the phenotypic values for the different environments. (JPG 7017 kb) [file 12864_2016_2875_MOESM5_ESM.jpg]

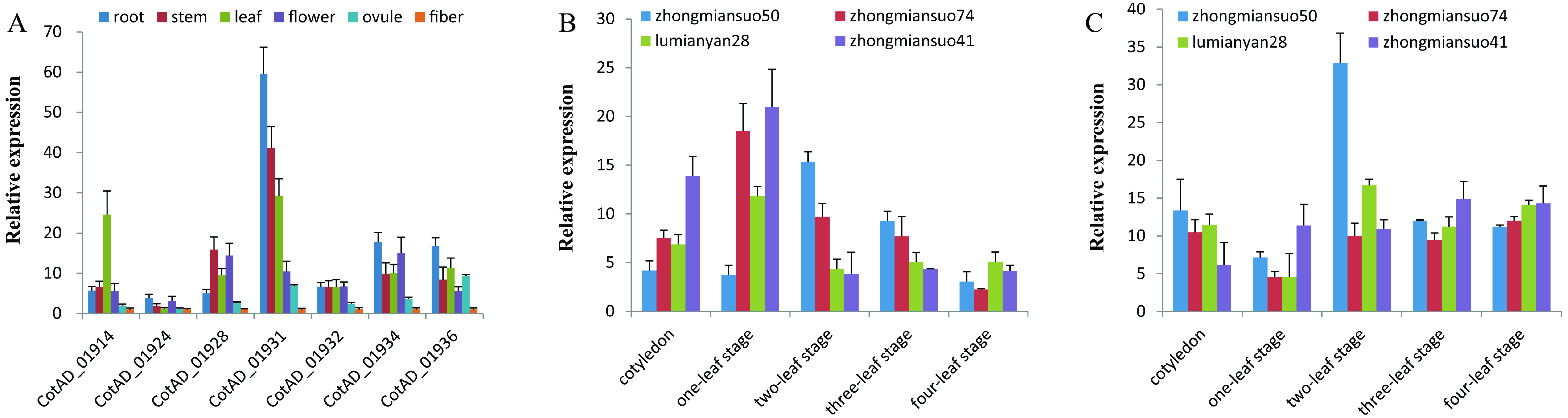

Supplement: Additional file 6: Figure S5. — Expression levels of several candidate genes that potentially underlie early maturity. (JPG 3083 kb) [file 12864_2016_2875_MOESM6_ESM.jpg]

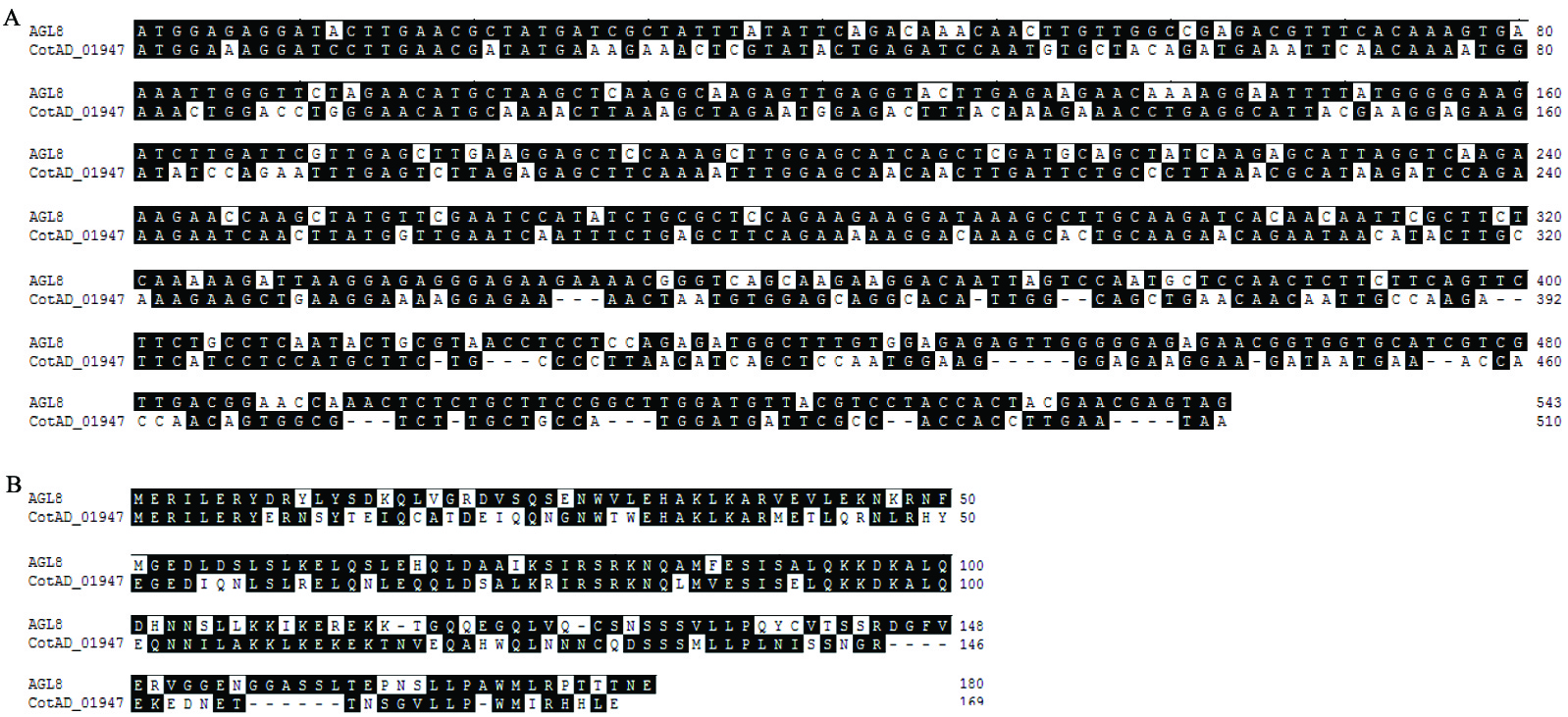

Supplement: Additional file 7: Figure S6. — Structure and identity of the MADS-box gene homolog CotAD_01947. (JPG 1502 kb) [file 12864_2016_2875_MOESM7_ESM.jpg]
